# The In Vitro Impact of Isoxazole Derivatives on Pathogenic Biofilm and Cytotoxicity of Fibroblast Cell Line

**DOI:** 10.3390/ijms24032997

**Published:** 2023-02-03

**Authors:** Urszula Bąchor, Adam Junka, Malwina Brożyna, Marcin Mączyński

**Affiliations:** 1 Department of Organic Chemistry and Drug Technology, Faculty of Pharmacy, Wroclaw Medical University, 50-556 Wroclaw, Poland; 2 Unique Application Model Laboratory, Department of Pharmaceutical Microbiology and Parasitology, Faculty of Pharmacy, Wroclaw Medical University, 50-556 Wroclaw, Poland

**Keywords:** isoxazole, Michael addition, Passerini reaction, anti-bacterial activity, cytotoxicity, antiseptics

## Abstract

The microbial, biofilm-based infections of chronic wounds are one of the major challenges of contemporary medicine. The use of topically administered antiseptic agents is essential to treat wound-infecting microorganisms. Due to observed microbial tolerance/resistance against specific clinically-used antiseptics, the search for new, efficient agents is of pivotal meaning. Therefore, in this work, 15 isoxazole derivatives were scrutinized against leading biofilm wound pathogens *Staphylococcus aureus* and *Pseudomonas aeruginosa*, and against *Candida albicans* fungus. For this purpose, the minimal inhibitory concentration, biofilm reduction in microtitrate plates, modified disk diffusion methods and antibiofilm dressing activity measurement methods were applied. Moreover, the cytotoxicity and cytocompatibility of derivatives was tested toward wound bed-forming cells, referred to as fibroblasts, using normative methods. Obtained results revealed that all isoxazole derivatives displayed antimicrobial activity and low cytotoxic effect, but antimicrobial activity of two derivatives, 2-(cyclohexylamino)-1-(5-nitrothiophen-2-yl)-2-oxoethyl 5-amino-3-methyl-1,2-oxazole-4-carboxylate (**PUB9**) and 2-(benzylamino)-1-(5-nitrothiophen-2-yl)-2-oxoethyl 5-amino-3-methyl-1,2-oxazole-4-carboxylate (**PUB10**), was noticeably higher compared to the other compounds analyzed, especially **PUB9** with regard to *Staphylococcus aureus*, with a minimal inhibitory concentration more than x1000 lower compared to the remaining derivatives. The **PUB9** and **PUB10** derivatives were able to reduce more than 90% of biofilm-forming cells, regardless of the species, displaying at the same time none (**PUB9**) or moderate (**PUB10**) cytotoxicity against fibroblasts and high (**PUB9**) or moderate (**PUB10**) cytocompatibility against these wound cells. Therefore, taking into consideration the clinical demand for new antiseptic agents for non-healing wound treatment, **PUB9** seems to be a promising candidate to be further tested in advanced animal models and later, if satisfactory results are obtained, in the clinical setting.

## 1. Introduction

The non-healing wound is referred to as the discontinuity of skin and subcutaneous tissue, which does not heal in an orderly set of stages and in a predictable amount of time. It is estimated that procedures related with management and treatment of chronic wounds consume approximately 1–3% of European Union public health budgets [1]. The presence of non-healing wounds significantly increases risk of serious health loss and, in case of wound infection, even death of the patient. The non-healing wounds are frequently colonized by differentiated consortia of bacteria, of whom Gram-positive *Staphylococcus aureus* and Gram-negative *Pseudomonas aeruginosa* should be distinguished because of their particular tendency to spread in the wound bed and to cause local, biofilm-based infections, which may develop into the life-threatening systemic forms [2]. In turn, fungi, such as yeast-like *Candida albicans*, are less frequently isolated from the non-healing wounds. Nevertheless, they are an important component of so called mixed (duo- or multi-species) biofilms in this niche; therefore, their potential impact on the process of wound healing should not be neglected [3]. The present treatment algorithms for critically colonized/infected non-healing wounds consist of wound bed debridement (of surgical, chemical/enzymatical or biological nature), use of modern dressings and application of locally administered antimicrobials referred to as the antiseptics [4]. The last-mentioned measure, used for both prophylactic and treatment purposes, are compounds of rapid antimicrobial action and preferentially of low or none cytotoxicity toward cells forming the wound bed, i.e., fibroblasts and keratinocytes [5]. Nevertheless, in recent years, the phenomenon of increased microbial tolerance and/or resistance toward many of the most widespread antiseptics occurred, just to mention cases of chlorhexidine and octenidine dihydrochloride [6,7]. Because use of antiseptics in the treatment of infected non-healing wounds is a critical factor with regard to clinical success (wound closure), presently the increasing search for new compounds, which may replace or improve activity of existing antiseptics, can be observed. One of the examples of such compounds are bioactive small molecules of a molecular weight <900 Da. These low-mass molecules make up presently 90% of pharmaceutical drugs (including such common drugs as aspirin or insulin). Thanks to their low size, such drugs can be administered orally and are able to pass through cell membranes and reach intracellular targets [8]. 

Much attention has been paid to the synthesis of heterocycles containing both nitrogen and oxygen due to their broad spectrum of biological and pharmacological activities. Among the wide range of pharmacologically important heterocycles, isoxazoles play a significant role in the field of medicinal chemistry. This heterocycle is considered as an important class of compounds in medicinal chemistry because of its wide spectrum of targets and varied biological application such as antimicrobial [9], antiviral [10], anticancer [11], anti-inflammatory [12], immunomodulatory [13], anticonvulsant [14] or antidiabetic properties [15]. The key feature of these heterocycles is that they exhibit the typical properties of an aromatic system but the same contain a weak nitrogen-oxygen bond that can be easily cleaved under certain reaction conditions. Thus, isoxazoles are very useful substrates since the ring system stability allows the derivatization of substituents to give functionally complex derivatives. Emerging research interest on the isoxazole moiety results from fact that this moiety is a common synthetic building block in searching for new compounds exhibiting antimicrobial and antifungal activities [16,17]. Synthesis of isoxazole derivatives is widely carried out through different methods such as condensation, cyclomerization or cycloaddition. Thus, the synthesis of new isoxazole derivatives is a very attractive aspect in the research and development field for both medicinal and organic chemistry. Due to these facts as well as to its relatively easy synthesis, isoxazole rings have become an object of our interest. Inspired by these facts we planned to synthesize some more derivatives of isoxazoles using different methods of functionalization such as Passerini multicomponent reaction and Michael addition.

Of note, the presence of an isoxazole moiety translates frequently into antimicrobial potential of such low-mass molecules as antibiotics: sulfisoxazole, cloxacilin, dicloxacilin or sulfamethozaole [18]. Isoxazole derivatives obtained earlier by our team also possessed a number of biological activities, including those of immunosuppressive [19,20,21,22], antiviral [23] or anticancer character [24]. It is well-recognized that conjugation of a small molecule to the isoxazole core offers a possibility to obtain new derivatives of biological activity [25]. As an example, maleimides are one of the most widely used functional groups for 1,4-conjugate addition. Their chemistry is widely used in the site-selective modification of thiol- and amine-containing compounds [26]. Although some types of nucleophiles can be involved in such a conjugation, ubiquitous heteronucleophiles (e.g., thiols, amines, and alcohols) are used the most frequently and, therefore, they are the most scrutinized and developed. For a number of years, these conjugate additions were limited to organic chemistry. Nevertheless, in the last few decades they have been also notably used in bioorganic and polymer chemistry for bioconjugation reactions and step-growth polymerizations, respectively, thanks to their excellent efficiency and ambient reactivity [27]. The α,β-unsaturated bond presented in maleimides allows a process of easy functionalization. Moreover, the delocalization of the double bonds (two carbonyl groups and one double bond in the ring) gives maleimides electron acceptor and dienophile characteristics [28]. 

**Figure 1:**
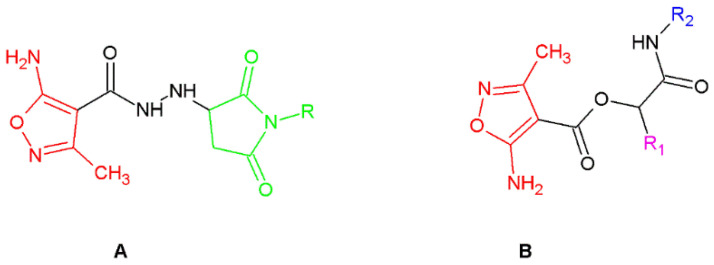
Designed isoxazole derivatives ((**A**) MAL1-5 series; (**B**) **PUB1-10** series: R1-aldehyde/ketone residue; R2-isocyanide residue).

Thus, click nucleophilic conjugate addition via aza-Michael reaction has become one of the ways to obtain a series of new isoxazole derivatives in this work (**MAL1-5** series) (Figure 1). In these reactions, we used 5-amino-3-methyl-isoxazole-4-carbohydrazide as a substrate [29,30]. We have employed this compound because this isoxazole derivative has attracted much attention due to its known biological activity [31]. According to the earlier investigation, this compound exhibits immunomodulatory properties, which makes it useful in further research and chemical modification [32,33]. Because the different approaches of modification of substituents in position 4 of 5-amino-3-methyl-isoxazole-4-carboxylic acid allow to obtain derivatives of different biological activity [34], we decided to employ this unnatural amino acid to a Passerini three-component reaction (**PUB1-10** series) (Figure 1). This type of reaction is the oldest isocyanide-based multicomponent reaction employing isocyanide (R_2_), aldehyde/ketone (R_1_) and carboxylic acid to give acyclic depsipeptides [35]. This type of peptide contains one or more ester bonds in addition to the amide bonds, and has become important lead structures in the development of new synthetic drugs. One example of this class of compounds is enniatins, which are cyclohexadepsipeptides with a range of biological activities, including those of antibiotic and cytotoxic character [36].

Therefore, the aim of this work was to scrutinize obtained isoxazole derivatives with regard to their antimicrobial (including antibiofilm) potential, as well as their impact on fibroblast cell lines. Moreover, following the recent trends of fortification of wound dressings with antimicrobials other than antibiotics or clinically used antiseptics, we introduced the isoxazole derivatives to the experimental dressing made of polymeric bacterial cellulose and assessed its activity against biofilms [37,38,39]. Such a goal fits in the current trend of research aiming for introduction of new agents which can be considered an alternative for already existing antiseptic agents used in the prophylaxis and treatment of critically colonized non-healing wounds.

## 2. Results and Discussion

### 2.1. Chemistry

**Figure 2:**
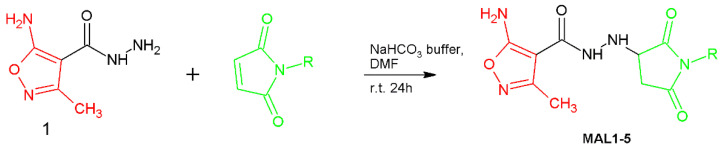
Synthesis of isoxazole linked maleimide conjugates (**MAL1-5**).

A series of isoxazole derivatives referred to in this work as the **PUB** series (**PUB1-8**) were obtained by our team earlier and their effects on phytohemagglutinin A (PHA)-induced proliferation of human peripheral blood mononuclear cells (PBMC), production of tumor necrosis factor alpha (TNFα) in human whole blood cultures stimulated with lipopolysaccharide (LPS) and two-way mixed lymphocyte reaction (MLR) of PBMC were investigated [25]. These compounds as well as **PUB9** and **PUB10** were obtained via Passerini three-component reaction by using methods published previously [25]. Briefly, *N′*-substituted 5-amino-*3*-methyl-isoxazole-4-carbohydrazide derivatives were synthesized using click nucleophilic conjugate addition via aza-Michael reaction according to Figure 2 presented below.

Maleimide is an unsaturated imide that forms pyrrole-type ring structures (1*H*-pyrrole-2,5-diones). The double bond presented in maleimides is highly electron-deficient due to the presence of two carbonyl groups, which consequently makes maleimides highly reactive and undergo nucleophilic addition reaction, called Michael addition, and is a type of “click chemistry”. The Michael addition with a primary amine is faster and reaches a higher conversion rate than its reactions with thiols [19]. A new series of *N*-substituted hydrazide derivatives was obtained by reacting 5-amino-3-methyl-isoxazole-4-carbohydrazide with different commercially available maleimides. This isoxazole derivative exhibits the unique reactivity, which is due to the selectivity of reaction of the amino groups present in this compound. The maleimides react with the amine group presented in hydrazide moiety, whereas we did not observe any product of reaction between the maleimide and NH_2_ group derived from the isoxazole ring. This unique property of the amino group attached to the isoxazole ring has been examined by our team in different kinds of reaction and as a result it has been shown that it remains unreactive in almost types of reactions [40,41]. The reaction is pH selective and favors primary amines in more alkaline conditions, but it unfortunately also increases the rate of hydrolysis of the maleimide group, resulting in a maleamic acid which is a non-reactive form of maleimide. Therefore, one of the most important tasks in our work was the optimalization of the reaction conditions to avoid this synthetic problem. In the course of the conducted chemical synthesis, it turned out that the most suitable pH for carrying out this reaction was pH = 7.8, which made it possible to use a bicarbonate buffer. By employing a slight excess of 5-amino-3-methyl-isoxazole-4-carbohydrazide, we ensured the full consumption of each of the used maleimides, consequently improving the yields of isolated products of conjugation to obtain a new series of isoxazole derivatives (series **MAL1-5**) (Figure 2). 

### 2.2. Synthesis and Structural Characterization

The synthesis and characterization of compounds **PUB1-8** has been published previously [25]. Compounds **PUB9** and **PUB10** were obtained according to the method described previously and purified by crystallization from methanol (**PUB10**) and by column chromatography (**PUB9**) with silica gel 230–400 mesh (60 Å); mobile phase: ethyl acetate/ chloroform = 3/7 (*v*/*v*), sample dissolved in chloroform. Structures of new compounds and their spectral analysis are shown in the Supplementary Material (Figures S16–S21).

#### 2.2.1. 2-(cyclohexylamino)-1-(5-nitrothiophen-2-yl)-2-oxoethyl 5-amino-3-methyl-1,2-oxazole-4-carboxylate (**PUB9**)

30 % yield; m.p. 199–200 °C, beige solid. ^1^H NMR (300 MHz, DMSO-*d*_6_) δ (ppm): 1.01–1.34 (m, 5H), 1.50–1.80 (m, 6H). 2.25 (s, 3H, CH_3_ group of isoxazole ring), 4.09 (q, J = 5.3 Hz, 1H), 6.34 (s, 1H), 7.26–7.31 (d, J = 4.22 Hz, 1H), 7.91 (bs, 2H, NH_2_ group from isoxazole ring), 8.05 (d, J = 4.3 Hz, 1H), 8.42 (d, J = 7.8 Hz, 1H). ^13^C NMR (75 MHz, DMSO-*d*_6_) δ (ppm): 12.43, 24.70, 24.79, 25.52, 31.13, 32.34, 32.47, 39.15, 39.43, 39.71, 39.98, 40.26, 40.54, 40.81, 48.49, 49.05, 70.48, 84.42, 127.18, 129.91, 147.31, 161.31, 165.62, 172.65. ESI-MS: *m*/*z* calculated for formula C_17_H_20_N_4_O_6_S [M-H]^−^ 407.103, found 407.103.

#### 2.2.2. 2-(benzylamino)-1-(5-nitrothiophen-2-yl)-2-oxoethyl 5-amino-3-methyl-1,2-oxazole-4-carboxylate (**PUB10**)

20% yield; m.p. 201–202 °C, beige solid. ^1^H NMR (300 MHz, DMSO-*d*_6_) δ (ppm): 2.25 (s, 3H, CH_3_ groups of isoxazole ring), 4.29–4.35 (d, J = 5.8 Hz, 2H), 6.44 (s, 1H), 7.16–7.35 (m, 6H), 7.94 (bs, 2H, NH_2_ group from isoxazole ring), 8.04–8.08 (d, J = 5.5 Hz, 1H), 9.02 (t, J = 5.9 Hz, 1H). ^13^C NMR (126 MHz, DMSO-*d*_6_) δ (ppm): 12.52, 42.80, 70.74, 84.45, 127.38, 127.46, 127.60, 128.82, 129.93, 138.98, 146.86, 151.42, 159.37, 161.40, 166.90, 172.72. ESI-MS: *m*/*z* calculated for formula C_18_H_16_N_4_O_6_S [M-H]^−^ 415.072, found 415.076.

The novel target compounds (**MAL1-5**) were synthesized according to the procedure described below using 5-amino-3-methylisoxazole-4-carbohydrazide, which was obtained by a sequence of very efficient processes described in detail in the following literature entries [29,42,43]) and different commercially available *N*-substituted maleimides as starting materials.

#### 2.2.3. General Procedure for Preparation of a Series of Compounds (MAL1-5)

To a solution of 5-amino-3-methylisoxazole-4-carbohydrazide (**1**) (2.0 mmol) in DMF (5 mL) was added a solution of each *N*-maleimide (1.8 mmol) in 5 mL DMF with 2 mL of NaHCO_3_ buffer (8.4 mg NaHCO_3_ dissolved in 10 mL of water). The reaction mixture was stirred at room temperature for 24 h, then DMF was removed using a gentle stream of air. The final product was purified by crystallization from methanol. Spectral analysis of new compounds as well as their structures are shown in the Supplementary Material (Figures S1–S15).

#### 2.2.4. 5-amino-N′-(2,5-dioxopyrrolidin-3-yl)-3-methyl-1,2-oxazole-4-carbohydrazide (MAL1)

40% yield; m.p. 220–221 °C, orange solid. ^1^H NMR (300 MHz, DMSO-d_6_) δ (ppm): 2.21 (s, 3H, CH_3_ group of isoxazole ring), 2.56–2.90 (m, 2H), 3.90–4.04 (m, 1H), 5.42–5.76 (m, 1H), 7.25–7.62 (bs, 2H, NH_2_ group from isoxazole ring), 8.34–8.87 (d, 1H, J = 5.5 Hz), 10.97–11.42 (s, 1H). ^13^C NMR (75 MHz, DMSO-*d*_6_) δ (ppm): 11.54, 35.21, 58.91, 86.23, 157.25, 163.72, 171.02, 177.24, 178.03. ESI-MS: *m*/*z* calculated for formula C_9_H_11_N_5_O_4_ [M+H]^+^ 254.088, found 254.081.

#### 2.2.5. 5-amino-3-methyl-N′-(1-methyl-2,5-dioxopyrrolidin-3-yl)-1,2-oxazole-4-carbohydrazide (MAL2)

55% yield; m.p. 173–174 °C, orange solid. ^1^H NMR (300 MHz, DMSO-*d*_6_) δ (ppm): 2.19 (s, 3H, CH_3_ group of isoxazole ring), 2.54–2.64 (m, 1H), 2.82 (s, 3H, CH_3_ group of maleimide moiety), 2.85–2.92 (m, 1H), 3.95–4.06 (m, 1H), 5.62–5.78 (m, 1H), 7.41–7.48 (bs, 2H, NH_2_ group from isoxazole ring), 8.56–8.73 (d, 1H, J = 5.39 Hz). ^13^C NMR (126 MHz, DMSO-d_6_) δ (ppm): 11.68, 24.43, 34.22, 57.89, 86.62, 157.43, 163.88, 171.19, 176.09, 176.76. ESI-MS: *m*/*z* calculated for formula C_10_H_13_N_5_O_4_ [M+H]^+^ 268.104, found 268.098.

#### 2.2.6. 6-(3-{2-[(5-amino-3-methyl-1,2-oxazol-4-yl)carbonyl]hydrazinyl}-2,5-dioxopyrrolidin-1-yl)hexanoic acid (MAL3)

37% yield; m.p. 143–144 °C, white solid. ^1^H NMR (300 MHz, DMSO-*d*_6_) δ (ppm): 1.10–1.56 (m, 6H), 2.08–2.18 (m, 2H), 2.19 (s, 3H, CH_3_ group of isoxazole ring), 2.54–2.67 (m, 2H), 2.80–2.93 (m, 1H), 3.93–4.08 (m, 1H), 5.63–5.82 (m, 1H), 7.37–7.55 (bs, 2H, NH_2_ group from isoxazole ring), 8.56–8.70 (d, 1H, J = 4.91), 11.81–12.09 (bs, 1H, COOH group from maleimide moiety). ^13^C NMR (75 MHz, DMSO-d_6_) δ (ppm): 11.99, 24.45, 26.16, 27.34, 33.86, 34.27, 38.15, 58.00, 86.65, 157.66, 164.32, 171.54, 174.80, 176.31, 177.02. ESI-MS: *m*/*z* calculated for formula C_15_H_21_N_5_O_6_ [M+Na]^+^ 390.138, found 390.132.

#### 2.2.7. 5-amino-N′-(1-cyclohexyl-2,5-dioxopyrrolidin-3-yl)-3-methyl-1,2-oxazole-4-carbohydrazide (MAL4)

27% yield; m.p. 193–195 °C, white solid. ^1^H NMR (300 MHz, DMSO-*d*_6_) δ (ppm): 0.94–2.08 (m, 10H), 2.20 (s, 3H, CH_3_ group of isoxazole ring), 2.51–2.61 (m, 1H), 2.74–2.88 (m, 1H), 3.72–3.86 (m, 1H), 3.89–4.00 (m, 1H), 5.62–5.73 (m, 1H), 7.37–7.49 (bs, 2H, NH_2_ group from isoxazole ring), 8.53–8.73 (d, 1H, J = 5.4 Hz). ^13^C NMR (75 MHz, DMSO-*d*_6_) δ (ppm): 11.53, 24.81, 25.31, 28.36, 33.63, 50.53, 57.09, 86.15, 157.17, 163.85, 171.08, 175.79, 176.55. ESI-MS: *m*/*z* calculated for formula C_15_H_21_N_5_O_4_ [M+H]^+^ 336.166, found 336.161.

#### 2.2.8. 5-amino-N′-[1-(4-chlorophenyl)-2,5-dioxopyrrolidin-3-yl]-3-methyl-1,2-oxazole-4-carbohydrazide (MAL5)

45% yield; m.p. 217–220 °C, white solid. ^1^H NMR (300 MHz, DMSO-*d*_6_) δ (ppm): 2.22 (s, 3H, CH_3_ group of isoxazole ring), 2.69–2.87 (m, 1H), 2.95–3.19 (m, 1H), 4.10–4.27 (m, 1H), 5.81–5.94 (m, 1H), 7.25–7.37 (d, 2H, J = 8.11 Hz), 7.44–7.53 (bs, 2H, NH_2_ group from isoxazole ring), 7.51–7.64 (d, 2H, J = 8.28 Hz). ^13^C NMR (75 MHz, DMSO-*d*_6_) δ (ppm): 11.56, 34.23, 57.98, 86.21, 128.57, 128.96, 131.08, 132.72, 157.25, 163.87, 171.04, 174.77, 175.48. ESI-MS: *m*/*z* calculated for formula C_15_H_14_ClN_5_O_4_ [M+H]^+^ 364.081, found 364.072.

### 2.3. Biology

In the first line of biological line of experiments, the minimal inhibitory concentrations of analyzed compounds were evaluated using a microplate model towards Gram-positive and Gram-negative reference pathogens and yeast-like fungus (Table 1). Obtained results indicate that the synthesized compounds acted in a more efficient manner against *C. albicans* than against *S. aureus* and *P. aeruginosa*. Nevertheless, two of the compounds (**PUB9** and **PUB10**) displayed a few hundred times higher activity against *S. aureus* compared to the remaining compounds, maintaining at the same time comparable (to the remaining compounds) activity against *P. aeruginosa* and *C. albicans*.

**Figure 3:**
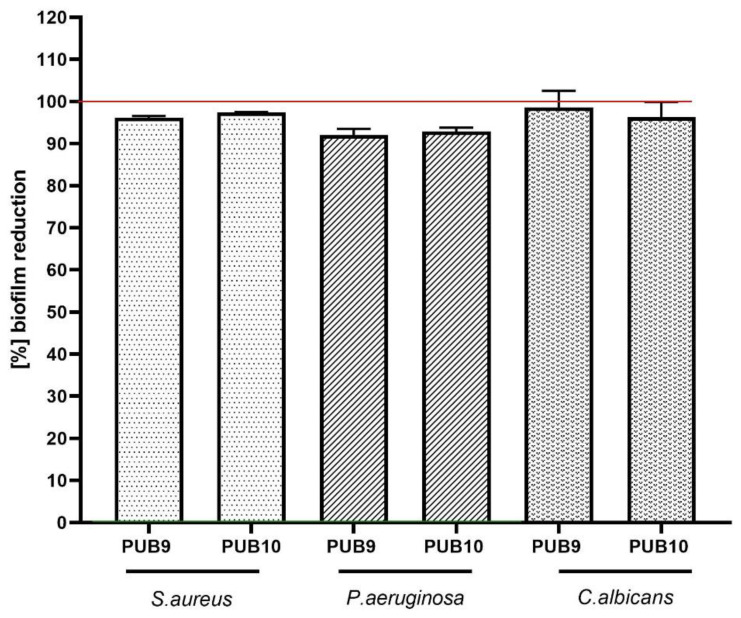
The [%] biofilm reduction of *S. aureus* 6538, *P. aeruginosa* 15,442 and *C. albicans* 103,231 displayed by **PUB9** and **PUB10** compounds at specific concentrations tested in microplate model. The red line shows level of 100% reduction while no reduction (0%, positive control of growth) was established for non-treated cells (maximal cellular growth).

Compounds **PUB9** and **PUB10** were thus selected for further analyses. The first of them included assessment of biofilm reduction in the microplate model. Obtained results indicate strong (above 90%) reduction in biofilm, regardless of the strain applied (Figure 3) in concentration range of 0.125–0.25 mg/mL.

**Figure 4:**
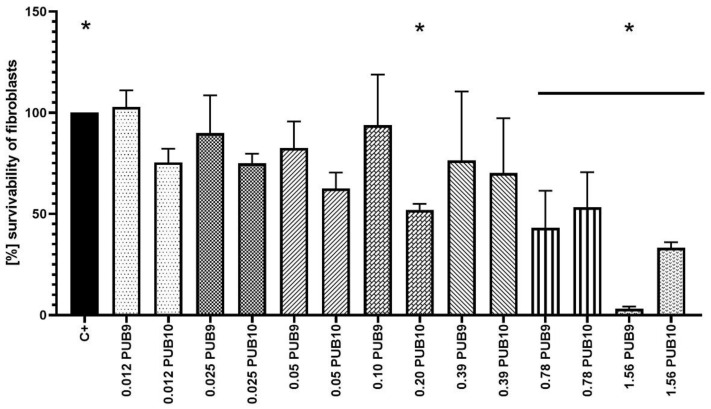
The survivability [%] of L929 fibroblasts exposed to the spectrum of concentrations of **PUB9** and **PUB10** compounds. The asterisks show significant (*p* > 0.05, ANOVA test with Tukey’s multiple comparison test) difference in survivability between control setting (non-treated cells, considered 100% growth) and cells treated with 0.78–1.56 mg/mL of **PUB9** or **PUB10** as well as 0.20 mg/mL of **PUB10**.

Next, the survivability of fibroblast cells (wound bed-forming cells) was analyzed after the exposure to the range of **PUB9** and **PUB10** compounds (Figure 4). In the case of **PUB9**, the advantageous results (cytotoxicity close to none) were obtained when 0.2 mg/mL of compounds was applied, while doubling of the compound’s concentration correlated with moderate level of cytotoxicity. In the case of **PUB10**, higher cytotoxicity level was observed towards fibroblasts (strong and very strong cytotoxicity when concentrations of 0.2 and 0.39 mg/mL, respectively, were applied). 

**Figure 5:**
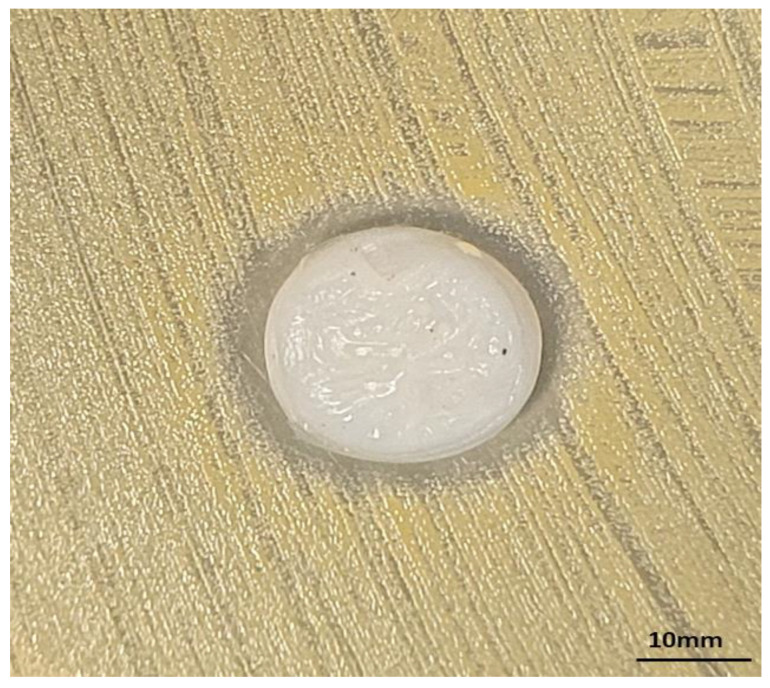
The inhibition of staphylococcal growth zone resulting from release of **PUB9** from BC carrier in modified disk-diffusion model. The diameter of BC carrier is 18 mm.

The results presented in Table 1, Figure 3 and Figure 4 allow obtaining data on the investigated compound’s in vitro activity against wound pathogens and also indicate in which concentration the specific compound does not exert (or exert at the acceptable level) cytotoxicity against fibroblast cells. In the next step, another two methods assessing the impact of antimicrobials against different types of microbial consortia were performed. In the first of them, referred to as the modified disk-diffusion model, the only full inhibition of microbial growth (equal 4 mm) was observed when **PUB9** against *S. aureus* was applied (Figure 5).

In the case of **PUB10**, the impact was also seen only against *S. aureus*; however, within the zone of growth inhibition (equal 2 mm), distinctive microbial colonies were also observed. Therefore, it was decided to apply another experimental model to get better insight on possible activity of **PUB9** and **PUB10** against biofilm. Using a modified Antibiofilm Dressing Activity Measurement model, the reduction resulting from activity of **PUB9** and **PUB10** was assessed. In the case of **PUB9**, the [%] reduction values were 99 ± 0.1%; 99 ± 0.1%, 90.7 ± 2.4% for *S. aureus*, *C. albicans* and *P. aeruginosa*, respectively, when 0.25 mg/mL of compound was applied. In case of exposure to 0.25 mg/mL of **PUB10**, the respective values were: 72.8 ± 0.04%; 69.8 ± 0.11% and 77.7 ± 2.4%. 

**Figure 6:**
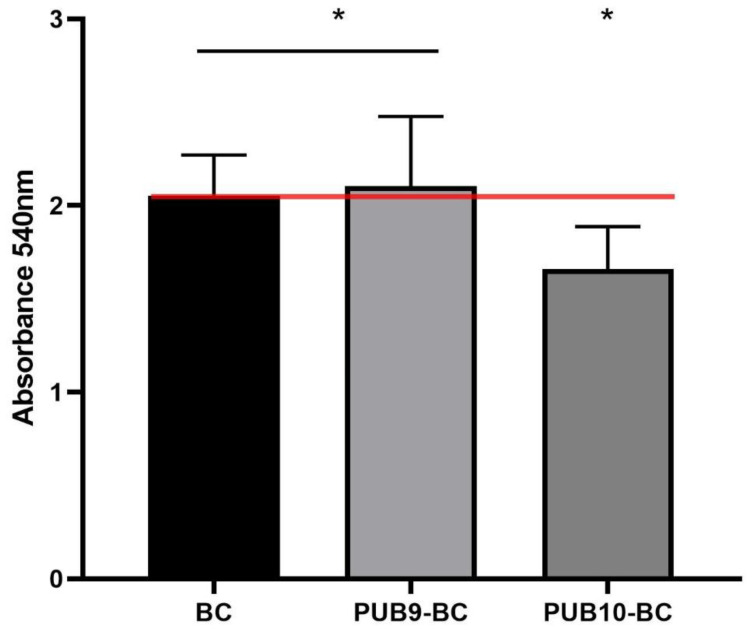
Comparison of cytocompatibility of isoxazole derivatives-fortified cellulosic carriers: **PUB9-BC** and **PUB10-BC** compared to control setting (cellulose carrier containing no isoxazole derivative) toward fibroblast cell line. Asterisks indicate significant differences (*p* < 0.05, ANOVA Test with Tukey’s multiple comparison test) between BC vs. **PUB10-BC** and **PUB9-BC** vs. **PUB10-BC**. The red line indicates the maximal potential cellular growth (of non-fortified cellulosic carrier).

In turn, the cytocompatibility tests of **PUB9**- and **PUB10**-containing BC carriers toward the fibroblast cell line corresponded to the obtained data on fibroblasts’ cytotoxicity, i.e., **PUB9-BC** displayed excellent cytocompatibility while **PUB10-BC** displayed the moderate cytocompatibility (Figure 6) compared to the biocompatibility of native BC.

The increasing frequency of non-healing wounds occurrence in the developed populations of the Western hemisphere accelerates the search for efficient algorithms in treatment of these disease entities. Infection is considered one of the most dangerous complications of non-healing wounds, and may lead even to the patient’s death. The local application of antiseptic agents is one of the pillars of modern wound treatment [44]. Nevertheless, continuous and prolonged exposure of infected wounds to antiseptics starts to induce processes analogical to those observed during application of antibiotics, namely to the rise of microbial tolerance and resistance [45]. Therefore, new classes of antiseptic agents are needed to overcome the threat of bacterial resistance and to provide wound professionals an effective tool in the fight against microbial biofilms colonizing and infecting wound beds. 

The isoxazole derivatives seem to be promising candidates in this regard, thanks to their relatively easy synthesis, chemical stability, low toxicity and good bioactivity at low doses. Although numerous methods for isoxazole-containing drugs have been developed, due to their wide activity, there is still a strong need to synthesize new derivatives containing this valuable moiety with increased chemical and pharmacological properties and test their biological activity. The current research focused on the antimicrobial (including antibiofilm) activity of isoxazole derivatives and, simultaneously, on their impact on wound bed-forming cells, referred to as the fibroblasts. The general aim was thus to investigate potential applicability of isoxazole derivatives in the character of drugs for infected wound care. While the MIC evaluation and modified disk diffusion methods served, to a certain extent, as the predictors of biofilm prevention potential, the MBEC and A.D.A.M. techniques showed the performance of isoxazole derivatives in the character of drugs for biofilm treatment. In turn, tests performed on fibroblasts provided insight on potential interaction of derivatives with wound cells, especially their ability to adhere and proliferate. 

The previous research on isoxazoles indicates that the substitution of various groups on the isoxazole ring imparts different activity. It was observed that the introduction of the thiophene moiety to the isoxazole ring increases its antimicrobial activity [46]. Previously, in vitro antibacterial and antifungal activity against *S. aureus, B. subtilis, E. coli, P. aeruginosa, A. niger* and *C.albicans* of 4,5-dihydro-5-(substitutedphenyl)-3-(thiophene-2-yl)isoxazole derivatives using the disc diffusion method was investigated by Gautam and Singh [47]. The obtained results clearly demonstrated high impact of the thiophene moiety on the analyzed biological activity. Another useful result was obtained by RamaRao et al. [48], who synthesized heteroarylisoxazoles and evaluated them for antibacterial activity against *E. coli*, *S. aureus* and *P. aeruginosa*. It was found that the isoxazoles substituted with the thiophenyl moiety displayed significant activity. The fact that the thiophene nucleus has been recognized as an important moiety in the synthesis of heterocyclic compounds with promising pharmacological characteristics prompted us to investigate such phenomena in the case of new isoxazole derivatives in the form of Passerini reaction products. In this work, 15 isoxazole derivatives were scrutinized with regard to their antimicrobial effect displayed against *S. aureus*, *P. aeruginosa* and *C. albicans*. The reason standing behind the choice of aforementioned species was not only different structure and composition of their cell wall, but also the fact that both *S. aureus* and *P. aeruginosa* belong to the pathogens being frequent etiological factors of non-healing wounds, while *C. albicans* is a component of mixed wound biofilms. Obtained results (Table 1) indicate that all tested compounds were able to inhibit growth of *S. aureus*, *P. aeruginosa* and *C. albicans* within the tested range of concentrations in the basic microplate model. Of note, in the case of 11/15 tested compounds there were no differences in Minimal Inhibitory Concentrations against *S. aureus* and *P. aeruginosa* displayed by a particular compound or these differences were on the level of just one geometric dilution, which may be related with the limitation of the method itself [49]. In turn, in the case of two compounds (**PUB9** and **PUB10**) the MIC value against *S. aureus* was more than 1000 and 260 times lower, respectively, than against *P. aeruginosa*. With regard to the chemical composition, **PUB9** and **PUB10** differed from the other analyzed compounds mainly by the presence of a thiophene ring. It was speculated by another research team that the presence of the positive charge of donor S atoms in the heteroaromatic ring was responsible for damage of the microbial cell wall/membrane, potential leakage of cytoplasmatic content to the environment and eventual cellular damage [50]. 

In the second line of investigation, the most promising isoxazole derivatives (**PUB9** and **PUB10**) were scrutinized with regard to their antibiofilm properties (Figure 3). The level of biofilm eradication above 90% was achieved when higher concentrations of compounds were applied (compared to the MIC values presented in Table 1). At the same time there were no significant differences in the value of reduction in biofilms formed by applied species (*p* < 0.05), which suggests the dose-dependent mode of action of tested isoxazole derivatives and the mechanism of biofilm eradication based on killing of microbial cells rather than destruction of biofilm matrix [51].

Nevertheless, such an observation confirms the well-recognized higher tolerance of biofilms against antimicrobials, compared to the tolerance displayed by their planktonic (non-adhered) counterparts [52]. Interestingly, the difference between concentrations necessary to reach MIC value vs. concentrations required to reach >90% of biofilm eradication was noticeably lower in the case of *P.aeruginosa* or *C.albicans* (two to four times) than in the case of *S.aureus* (ca. 2000–4000 times). Such a phenomenon may be related with the hypothetical mechanism of action of **PUB9** and **PUB10**, i.e., the inhibition of GTPase activity and bacterial cell division, which cause bactericidal effects [53]. The appropriate candidate for a wound antiseptic should display not only the sufficient antimicrobial (antibiofilm) activity, but also should not exert the harmful effect on the wound bed cells, such as fibroblasts [54]. Therefore, in the next analysis (Figure 4), the survivability of fibroblast cell line exposed to **PUB9** and **PUB10** activity was performed for the spectrum of concentrations, including these which proved to be sufficient to eradicate the pathogens in planktonic and biofilm forms (Table 1, Figure 3). The obtained results indicated the concentration-dependent growth of cytotoxic effects displayed by both compounds. Nevertheless, it also occurred that in the concentration range 0.2–0.4 mg/mL, **PUB9** displays acceptable (low) level of cytotoxicity, while acceptable level of cytotoxicity was recorded for **PUB10** in the concentration range 0.012–0.025 mg/mL. The exact mechanism explaining why certain isoxazole derivatives containing a thiophene ring display a cytotoxic effect toward certain cell lines but not other types still needs to be elucidated [55]. Nevertheless, in the current research two derivatives of low cytotoxicity against fibroblast cells were found, which makes them promising candidates to be investigated more thoroughly. To further analyze the impact of **PUB9** and **PUB10** on microorganisms, the modified disk diffusion test was performed (Figure 5) [56], which confirmed higher antimicrobial effect exerted by **PUB9** than **PUB10**; the coherent results were obtained in case of the A.D.A.M. test performed, i.e., the higher antibiofilm activity of **PUB9** than **PUB10**. To our knowledge it is the first time the isoxazole derivatives were introduced to the BC carrier, which paves a way to their potential application not only as antiseptic agents in the form of liquid solutions, but also as an antimicrobial component of active dressings designed to treat chronic wounds. To get deeper insight on properties of isoxazole derivatives-containing BC, we performed a cytocompatibility test in vitro (Figure 6). Obtained results revealed that excellent cytocompatibility of BC, indicated by other research teams [57], was reduced moderately (but in a statistically significant manner) by addition of **PUB10**, but not the **PUB9** compound. It may be thus assumed that the **PUB9** compound can be considered a candidate designed for treatment purposes (eradication of already formed biofilm), while **PUB10** appears to be more suitable for prophylaxis purposes (the lower, non-cytotoxic concentration of **PUB10** may be applied against planktonic forms of microorganisms, which did not develop into biofilm structure yet). 

Conventional, clinically applied drugs used topically for the treatment of chronic wounds can be divided into antibiotics or antiseptics. Gentamycin is an example of an antibiotic allowed to be used topically in the treatment of chronic wounds. This antibiotic is provided in a collagen scaffold, referred to as a “gentamycin-sponge”, or in a viscous hydrogel carrier. It was previously demonstrated that during the first 60 min of application of the sponge, released gentamycin reached a concentration of 1000 mg/L and during the next 4–5 days after implantation, the antibiotic concentration was at the level of 300–400 mg/L [58,59]. The indicated concentration of gentamycin sufficient to eradicate staphylococcal or pseudomonal biofilm in vitro was between 100–500 mg/L (depending on model applied) [60]. Of note, the concentration of the most efficient isoxazole derivative analyzed in the current work (**PUB9**) against *S. aureus* and *P. aeruginosa* biofilm was 125 mg/L. Such a result shows potential of **PUB9** to be applied for treatment of chronic wounds with regard to antibiofilm activity (come pared to antibiotics). The other class of products used to treat/prevent infections of chronic wounds is referred to as the antiseptics. These are mainly liquid solutions containing antimicrobial substances. The modern antiseptics include such antimicrobial compounds as octenidine dihydrochloride, povidone iodine or hypochlorous acids [54]. Due to the various mechanisms of action, the concentrations of various antiseptics necessary to eradicate pathogenic biofilm, differ significantly. The concentration of octenidine dihydrochloride (used in the current study as control of method’s usability) sufficient to eradicate the in vitro staphylococcal and candida biofilm was 62.5 mg/L (Table S1, Supplementary Data). This concentration was two times lower (more favorable with regard to antimicrobial level) than the concentration of the aforementioned **PUB9** compound. In turn, with regard to the *P. aeruginosa* biofilm, **PUB9** acted more efficiently (in microtiter plate model) than octenidine dihydrochloride. Such a differentiation in antiseptics’ efficacy may be applied for personalized medicine of treatment of chronic wound infections (under condition of performance of appropriate microbiological diagnostics). On the other hand, the working concentration of another common clinical antiseptic, povidone iodine, is 7.5%, and such a concentration exceeds significantly the microbiologically efficient concentrations of both **PUB9** and octenidine dihydrochloride. Therefore, the dissertations on antimicrobial efficacy should be always combined with data on compounds’ cytotoxic effect/cytocompatibility. These parameters were favorable for **PUB9** as shown in Figure 4 and Figure 6. The combined data on antimicrobial effect and cytocompatibility indicate that the **PUB9** derivative is of potential to be tested further in direction of its application as a compound for chronic wound treatment.

We are aware of the specific disadvantages of this work, derived mainly from its preliminary character, i.e., focusing on such basic parameters of obtained isoxazole derivatives as antimicrobial effect or cytotoxicity and not on, for example, such potentially important aspects as stability of derivatives introduced to the BC carrier, genotoxicity or the analysis of potential to induce antimicrobial resistance. Nevertheless, out of 15 synthesized isoxazole derivatives, two of them (**PUB9** and **PUB10**) displayed satisfactory antimicrobial effects, maintaining at the same time, acceptable level of cytotoxicity (the certain level of this feature is displayed by virtually all antiseptics [41]. These satisfactory (at this stage of research) results justify undertaking other investigation steps, including among others, testing derivatives using a *Galleria mellonella* infection animal model, performance of above-mentioned stability testing and potential of derivatives to induce antimicrobial resistance using tests as presented recently by Li et al. [61]. Taking into consideration the clinical demand for new antiseptic agents for non-healing wound treatment, the compounds presented in this work seem to be promising candidates to be further tested in more advanced animal models and later, if the satisfactory results are obtained, in the clinical setting.

## 3. Materials and Methods

### 3.1. Chemistry

Commercially available reagents, i.e., triethyl orthoacetate, cyanoacetate, hydrazine monohydrate, *N*-substituted maleimides, aldehydes and isocyanides, were purchased from Sigma-Aldrich (Merck Group, Darmstadt, Germany) or TCI (Tokyo, Japan) and were used without further purification. Thin layer chromatography (TLC) was used to assess the progress and completion of reactions and was carried out using Alugram SIL G/UV 254 nm plates (Macherey-Nagel, Düren, Germany) and the developing system ethyl acetate/chloroform = 3/7 (*v*/*v*), and visualized by ultraviolet (UV) light at 254 nm (UV A. KRÜSS Optronic GmbH, Hamburg, Germany). Melting points were determined by uniMELT 2 apparatus (LLG, Meckenheim, Germany) and were uncorrected. ^1^H NMR and ^13^C NMR spectra were obtained in DMSO-*d*_6_ and recorded using a Bruker ARX 300 MHz spectrometer (using TMS as the internal standard). 

All ESI-MS experiments were performed on the LCMS-9030 qTOF Shimadzu (Shimadzu, Kyoto, Japan) device, equipped with a standard ESI source and the Nexera X2 system. Analysis was performed in the positive and negative ion mode between 100–3000 *m*/*z*. LCMS-9030 parameters were the following: the nebulizing gas was nitrogen, the nebulizing gas flow was 3.0 L/min, the drying gas flow was 10 L/min, the heating gas flow was 10 L/min, interface temperature was 300 °C, desolvation line temperature was 400 °C, detector voltage was 2.02 kV, interface voltage was 4.0 kV, collision gas was argon, mobile phase (A) was H_2_O + 0.1% HCOOH, (B) was MeCN + 0.1% HCOOH, and mobile phase total flow was 0.3 mL/min.

### 3.2. Microorganisms

For research purposes, three following reference strains from the American Type and Culture Collection (ATCC) were used: *Staphylococcus aureus* 6538, *Pseudomonas aeruginosa* 15,442 and *Candida albicans* 103,231.

### 3.3. Determination of Minimal Inhibitory Concentration Using Microtiter Plate Method

To determine the minimal inhibitory concentration (MIC) values of the tested compounds, the following steps were performed. Firstly, overnight cultures of the strains were prepared in TSB medium (Tryptic Soy Broth, Biomaxima, Lublin, Poland) medium. Next, 100 µL of TBS was added to all wells of 96-well plates (Jet Bio-Filtration Co. Ltd., Guangzhou, China) and 100 µL of the tested substances or DMSO (dimethyl sulfoxide, VWR Chemicals, Radnor, PA, USA) (used as a control of the solvent’s antimicrobial activity) was added to the first columns of the pates, and ten geometric dilutions were performed for each compound. Subsequently, 0.5 MacFarland of bacterial/fungal suspensions were established in 0.9% solution of sodium chloride (NaCl, Stanlab, Lublin, Poland) using a densitometer (Densilameter II Erba Lachema, Brno, the Czech Republic). The suspensions were then diluted 1000 times in TSB and 100 µL was poured to the compounds-containing wells. Control of microorganisms’ growth (suspensions in TSB) and control of medium sterility (medium only) were also prepared. The absorbance of the solution was measured at 580 nm using a spectrophotometer (Multiskan Go, Thermo Fisher Scientific, Vantaa, Finland) and the plates were incubated at 37 °C for 24 h with shaking at 350 rpm (Mini-shaker PSU-2T, Biosan SIA, Riga, Latvia). The absorbance was measured after the incubation at 580 nm. The MIC values against *C. albicans* were evaluated in the first well, where no visible growth was observed. To determine MIC values against bacteria, 20 µL of 1% (*w*/*v)* TTC (2,3,5-triphenyl-tetrazolium chloride, AppliChem Gmbh, Darmstadt, Germany) in TSB was added to all wells and the plates were incubated for 2 h in the same conditions. The MIC value against bacteria was assessed in the first well where no red color was observed. There were three repetitions for each compound. The octenidine dihydrochloride-based antiseptic (Schulke Mayr, Nordstadt, Germany) served as control of method’s usability.

### 3.4. Determination of Minimal Biofilm Eradication Concentration Using Microtiter Plate Model

The overnight cultures of the strains were prepared in TSB medium. Subsequently, 0.5 MacFarland of bacterial/fungal suspensions were established in 0.9% solution of sodium chloride using a densitometer. The suspensions were then diluted 1000 times in TSB. An amount of 100 µL of such suspension was poured to wells of the 96-well plate and incubated for 18 h/37 °C. After incubation, the medium was removed, leaving biofilm-forming cells only. Next, the 100 µL of sterile TSB was poured to each well of the plate. Subsequently, the geometrical dilutions of tested compounds were performed in the wells of the 96-well plate. The whole experimental setting was then incubated for 18 h/37 °C. Afterwards, 20 µL of 1% (*w*/*v*) TTC in TSB was added to all wells and the plates were incubated for 2 h in the same conditions. The MBEC value against bacteria was assessed in the first well where no red color was observed. In the case of *C. albicans*, 20 µL of 0.1% resazurin in TSB was added to each well and incubated for 3h in the same conditions. The MBEC value against *C. albicans* was assessed in the first well where blue color was observed. There were three repetitions performed for each compound and each compound’s concentration. The formula used to determine the biofilm eradication was as follows: 100%—(value of absorbance obtained from biofilm treated with analyzed compound/absorbance value obtained from biofilm treated with saline) × 100%. These experiments were done in three repeats. The octenidine dihydrochloride-based antiseptic (Schulke Mayr, Nordstadt, Germany) served as control of method’s usability. 

### 3.5. The Cytotoxicity Assay of Analyzed Compounds towards Fibroblast Cell Line In Vitro

The Neutral Red (NR) cytotoxicity assay was performed toward fibroblast (L929 ATCC, Manassas, VA, USA) in vitro cell cultures treated with the analyzed compounds in concentration equal to their MBEC (or MIC if MBEC was beyond tested range of concentrations) value according to ISO 10993: Biological evaluation of medical devices; Part 5: Tests for in vitro cytotoxicity; Part 12: Biological evaluation of medical devices, sample preparation and reference materials (ISO 10993–5:2009 and ISO/IEC 17025:2005). The analyzed compounds were suspended in the medium for fibroblast culturing (RPMI, Sigma-Aldrich, Darmstad, Germany) and left to dry at room temperature. Next, 150 μL of a de-stain solution (50% ethanol, 96%, 49% deionized water, 1% glacial acetic acid; POCH, Lublin, Poland) was introduced to each well. The plate was shaken vigorously in a shaker (MTS4, IKA-Labortechnik, Berlin, Germany) for 30 min until NR was extracted from the cells and formed a homogenous solution. Finally, the value of NR absorbance was measured spectrometrically using a microplate reader at a wave of 540 nm length. The absorbance value of NR-dyed cells not treated with medium containing analyzed compounds was considered 100% of the potential cellular growth (positive control of growth), while cells treated with 70% EtOH (POCH, Lublin, Polska) for 30 min were considered the control of method’s usability. All analyses were performed in 6 repeats.

### 3.6. The Synthesis and Purification of Bacterial Cellulose Carrier and Impregnation of Carrier with Tested Compounds

The *K. xylinus* strain DSM 46604, used to bio-fabricate the BC carrier, was cultivated in stationary conditions for 7 days /28 °C in a 24-well plate (VWR International, Radnor, PA, USA) using a Hestrin-Schramm (H-S) medium. To remove bacterial cells and media components, the BCs were purified in 0.1 M NaOH (POCH, Lublin, Poland) for 90 min/80 °C. Next, BC carriers were immersed in distilled water and incubated with shaking. During this process, the pH value was measured every 3 hrs, until it reached a neutral value. The wet BC carriers were weighted. These of weight equal 1000 ± 100 mg were transferred to the 24-well plate and immersed with 1 mL of solution containing analyzed compounds in a concentration equal to 2 × MIC. The plate was left for 24 h/8 °C. After this time, BC carriers were used for analyses described in the subsequent sections of Material and Methods (Section 3.7, Section 3.8 and Section 3.9).

### 3.7. The Analysis of Antimicrobial Efficacy of Analyzed Compounds Released from BC Carriers in Modified Disk-Diffusion Method

For control purposes, BC carriers introduced to 1 mL of octenidine dihydrochloride (antiseptic substance of confirmed antimicrobial activity, Schulke-Mayr, Nordstadt, Germany) or 0.9% NaCl were applied. Firstly, the *S. aureus*, *P. aeruginosa* or *C. albicans* of 0.5/0.8 McFarland density were spread over the Muller-Hinton or Sabouraud agar plates, respectively. Next, BC carriers soaked with tested compounds were placed on the plates. The whole setting was subjected to incubation at 37 °C for 24 h. After that, the growth inhibition zone (if occurred) was measured using a ruler. The tests were performed in triplicates.

### 3.8. The Detection of Antibiofilm Activity of Tested Compounds Using Modified Antibiofilm Dressing Activity Measurement (A.D.A.M.)

The method was described in detail in the earlier work of ours [62]. The exposure time was 24 h. After incubation of preformed biofilm with compounds, biofilm-containing agar disks were transferred to the fresh wells of a 24-well plate. Next, 1 mL of 0.1% saponin solution was subjected to vortex-mixing for 1 min to detach the biofilm cells from the agar surface. Serial dilutions of the obtained suspension in saline solution were performed and then cultured onto Sabouraud or M-H agar plates (for *C. albicans* and *S. aureus/P. aeruginosa*, respectively). The plates were incubated at 37 °C for 24 h. After incubation, the cfu number of grown colonies was counted. The formula used to determine the biofilm eradication was as follows: 100%—(value of cfu obtained from biofilm treated with cellulose carrier soaked with analyzed compound)/cfu obtained from biofilm treated with non-impregnated cellulose) × 100%. These experiments were done in three repeats. The octenidine dihydrochloride-based antiseptic (Schulke Mayr, Nordstadt, Germany) served as control of method’s usability.

### 3.9. The Cytocompatibility of Isoxazole-Fortified BC Carriers to Fibroblast Cell Lines

One mL of culturing medium containing fibroblast cell lines of density equal 1 × 105 were seeded on the BC carriers fortified with the appropriate concentrations of **PUB9** and **PUB10** (0.25 mg/L) and cultured for 72 h at 37 °C/5% CO_2_ in an incubator. Next, the procedures utilizing Neutral Red method were performed to assess the survivability of the cells. The medium for fibroblast growth was removed and 100 μL of the NR solution (40 μg/mL; Sigma-Aldrich, Dormstadt, Germany) was introduced to wells of the plate. Cells were incubated with NR for 2 h at 37 °C. After incubation, the dye was removed, wells were rinsed with phosphate buffer saline (PBS, Sigma Aldrich, Dormstadt, Germany) and left to dry at room temperature. Next, 500 μL of a de-stain solution (50% ethanol 96%, 49% deionized water, 1% glacial acetic acid; POCH, Lublin, Poland) was introduced to each well. The plate was shaken vigorously in a microtiter plate shaker (MTS4, IKA-Labortechnik, Berlin, Germany) for 30 min until NR was extracted from the cells and formed a homogenous solution. Finally, the value of NR absorbance was measured spectrometrically using a microplate reader (Multi-scan GO, Thermo Fisher Scientific, Waltham, MA, USA) at 540 nm. The absorbance value of dyed fibroblasts seeded on the BC carriers non-fortified with **PUB9** and **PUB10** was considered 100% of the potential cellular growth (positive control).

### 3.10. The Statistical Analysis

Statistical analyses were performed using GraphPad Prism 8.0. (GraphPad Software, San Diego, CA, USA). Normality of distribution was verified using Shapiro–Wilk’s test. To evaluate statistical significance, the ANOVA test with post hoc Dunnett’s multiple comparison (α = 0.05) was performed.

## Figures and Tables

**Table 1:** The Minimal Inhibitory Concentration [mg/mL] of tested compounds towards *S. aureus* 6538, *P. aeruginosa* 15442 and *C. albicans* 103231 tested in microplate model.

Minimal Inhibitory Concentration [mg/mL]			
	*S. aureus*	*P. aeruginosa*	*C. albicans*
**PUB1**	0.125	0.125	0.063
**PUB2**	0.125	0.125	0.063
**PUB3**	0.125	0.125	0.063
**PUB4**	0.25	0.125	0.063
**PUB5**	0.25	0.125	0.063
**PUB6**	0.125	0.125	0.063
**PUB7**	0.125	0.125	0.063
**PUB8**	0.125	0.125	0.063
**PUB9**	0.00012	0.125	0.063
**PUB10**	0.00024	0.063	0.02
**MAL1**	0.125	0.063	0.02
**MAL2**	0.125	0.063	0.63
**MAL3**	0.125	0.125	0.063
**MAL4**	0.125	0.063	0.063
**MAL5**	0.125	0.063	0.063

## Data Availability

All necessary data are presented in the manuscript and the raw data can be provided from the authors upon reasonable request.

## Supplementary Materials

The following supporting information can be downloaded at: https://www.mdpi.com/article/10.3390/ijms24032997/s1.
