# Supplementary material for: The In Vitro Impact of Isoxazole Derivatives on Pathogenic Biofilm and Cytotoxicity of Fibroblast Cell Line
Source: Int J Mol Sci. 2023 Feb 3;24(3):2997. doi: 10.3390/ijms24032997 (PMC9917413; doi:10.3390/ijms24032997)
Supplement: Supplementary file 1 [file ijms-24-02997-s001.zip › ijms-2129607-supplementary.pdf]

SUPPLEMENTARY MATERIAL

The Impact of Isoxazole Derivatives on Pathogenic Biofilm and Cytotoxicity of Fibroblast Cell Line

Urszula Bąchor [1], Adam Junka [2,*], Malwina Brożyna [2] and Marcin Mączyński [1]

[1] Department of Organic Chemistry and Drug Technology, Faculty of Pharmacy, Wroclaw Medical University, 50-556 Wroclaw, Poland
[2] Department of Pharmaceutical Microbiology and Parasitology, Unique Application Model Laboratory, Faculty of Pharmacy, Wroclaw Medical University, 50-556 Wroclaw, Poland
* Correspondence: adam.junka@umw.edu.pl; Tel.: +48-71-784-06-75

Figure S1. ^{1}H NMR spectrum of compound MAL1 2
Figure S2. ^{13}C NMR spectrum of compound MAL1 2
Figure S3. ESI-MS spectrum of compound MAL1 3
Figure S4. ^{1}H NMR spectrum of compound MAL2 3
Figure S5. ^{13}C NMR spectrum of compound MAL2 4
Figure S6. ESI-MS spectrum of compound MAL2 4
Figure S7. ^{1}H NMR spectrum of compound MAL3 5
Figure S8. ^{13}C NMR spectrum of compound MAL3 5
Figure S9. ESI-MS spectrum of compound MAL3 6
Figure S10. ^{1}H NMR spectrum of compound MAL4 6
Figure S11. ^{13}C NMR spectrum of compound MAL4 7
Figure S12. ESI-MS spectrum of compound MAL4 7
Figure S13. ^{1}H NMR spectrum of compound MAL5 8
Figure S14. ^{13}C NMR spectrum of compound MAL5 8
Figure S15. ESI-MS spectrum of compound MAL5 9
Figure S16. ^{1}H NMR spectrum of compound PUB9 9
Figure S17. ^{13}C NMR spectrum of compound PUB9 10
Figure S18. ESI-MS spectrum of compound PUB9 10
Figure S19. ^{1}H NMR spectrum of compound PUB10 11
Figure S20. ^{13}C NMR spectrum of compound PUB10 11
Figure S21. ESI-MS spectrum of compound PUB10 12

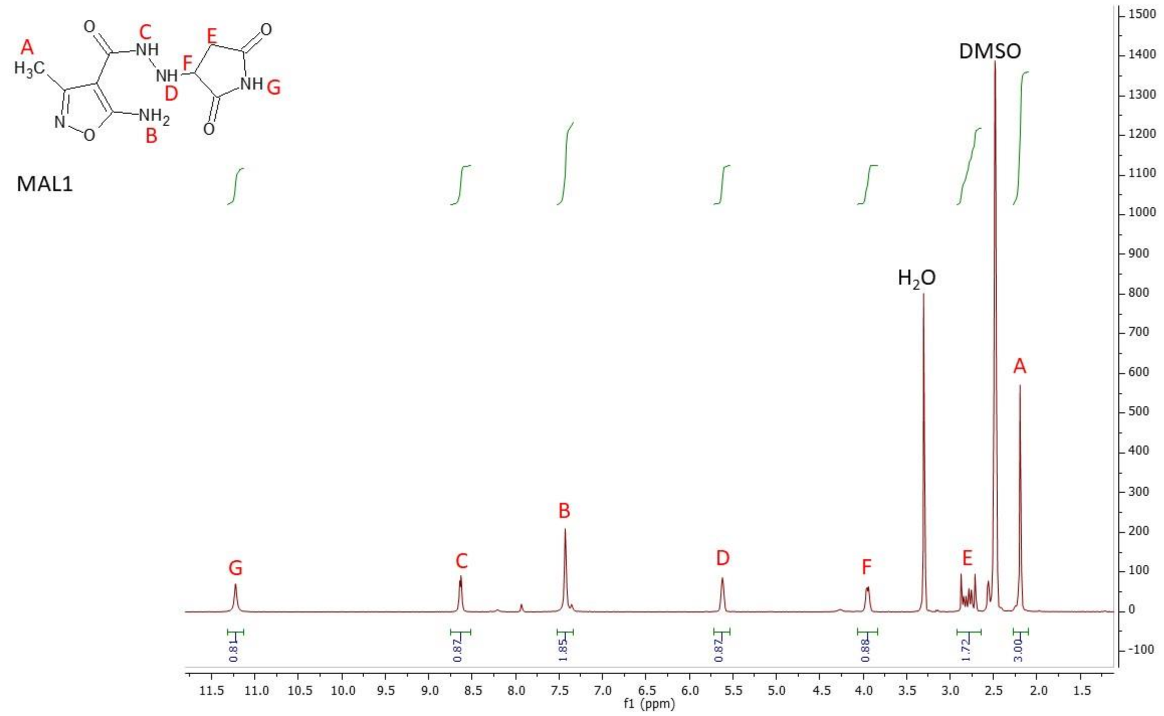

Figure S1. ^{1}H NMR spectrum of compound MAL1

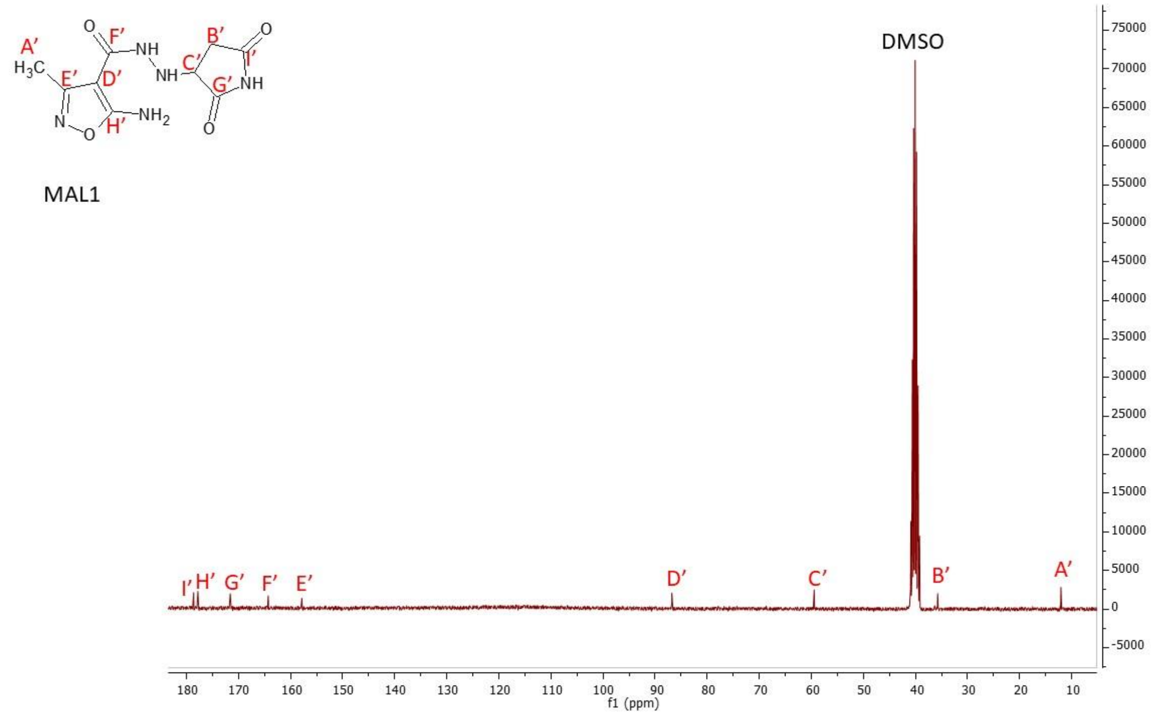

Figure S2. ^{13}C NMR spectrum of compound MAL1

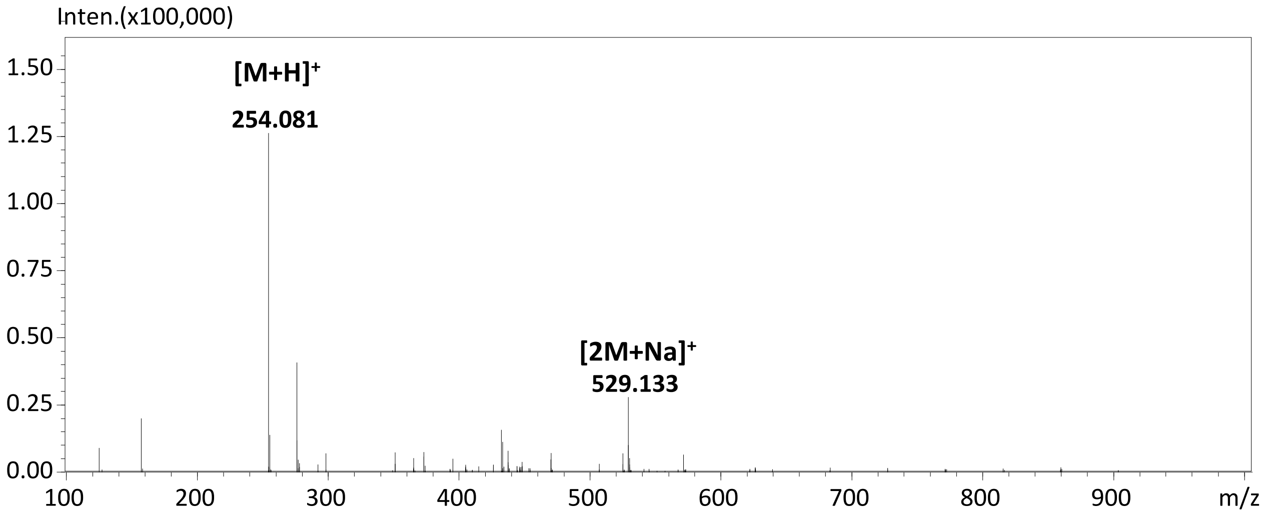

Figure S3. ESI-MS spectrum of compound MAL1

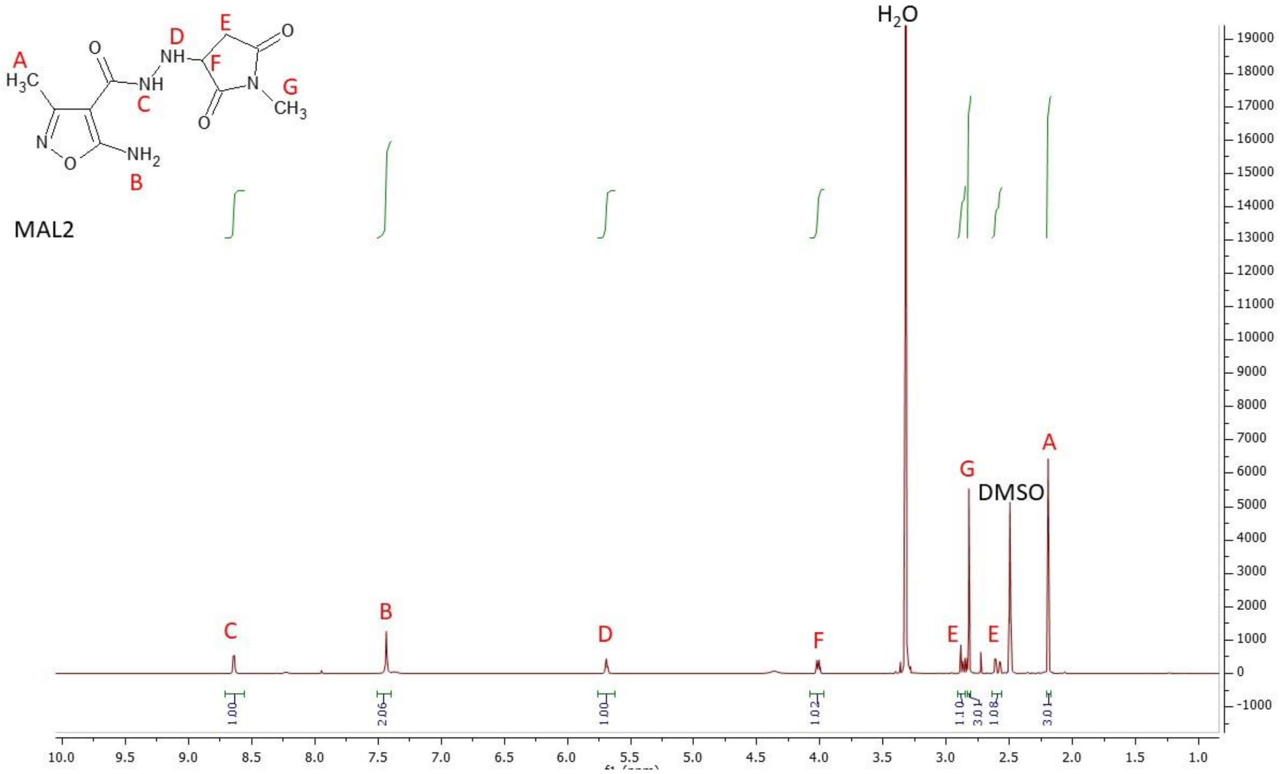

Figure S4. ^{1}H NMR spectrum of compound MAL2

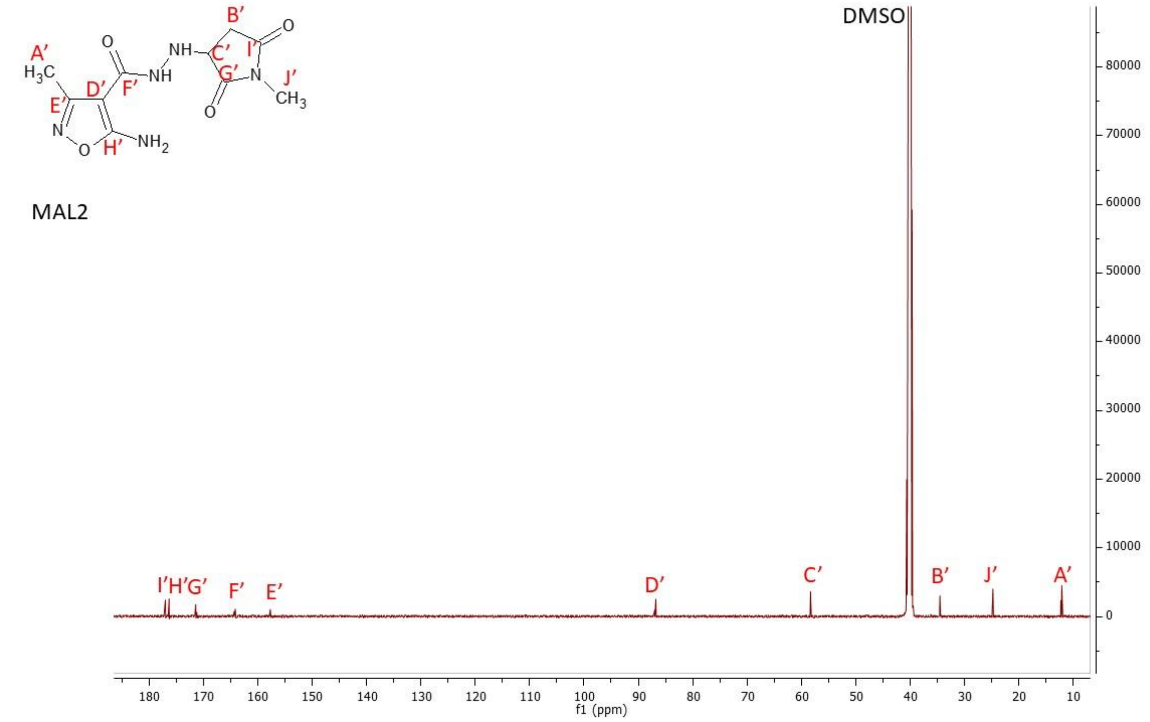

Figure S5. ^{13}C NMR spectrum of compound MAL2

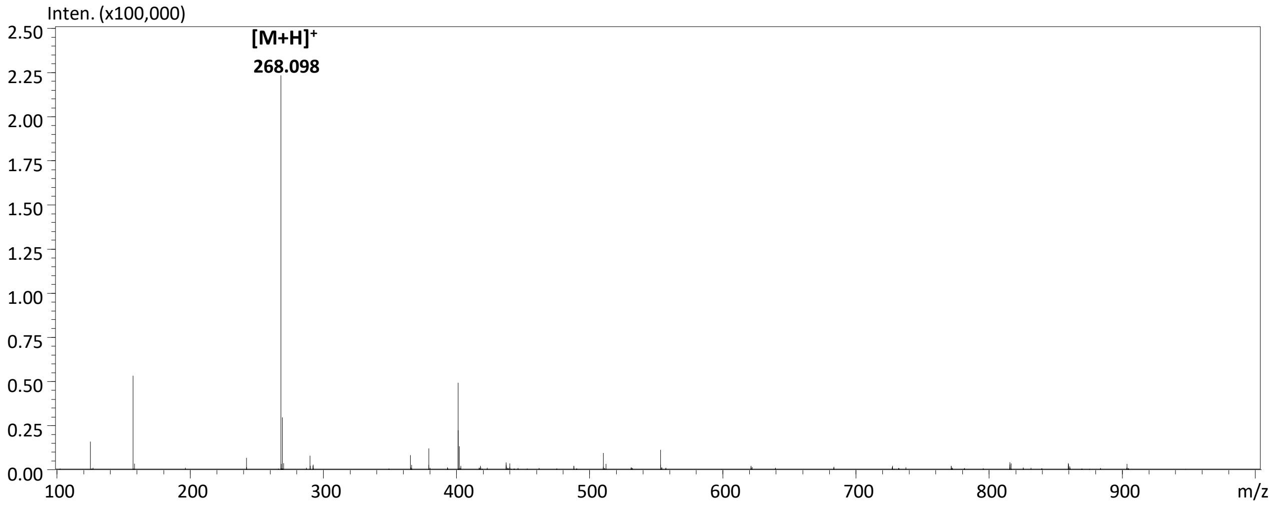

Figure S6. ESI-MS spectrum of compound MAL2

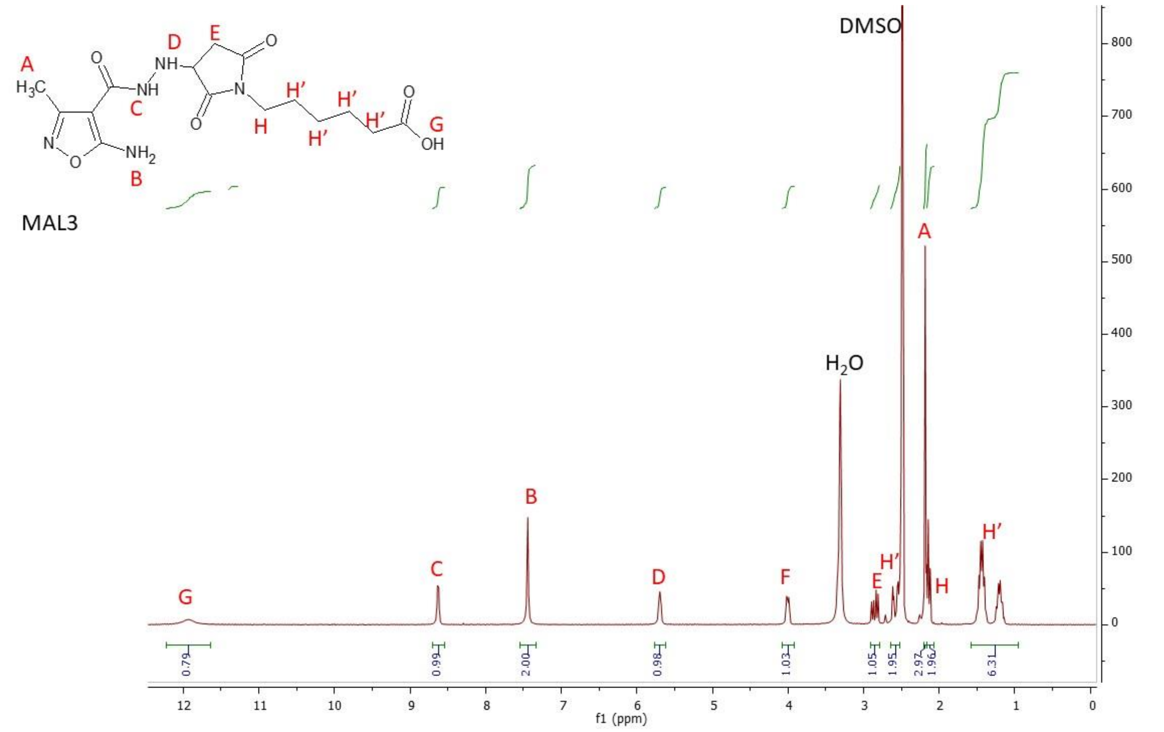

Figure S7. ^{1}**H NMR spectrum of compound MAL3**

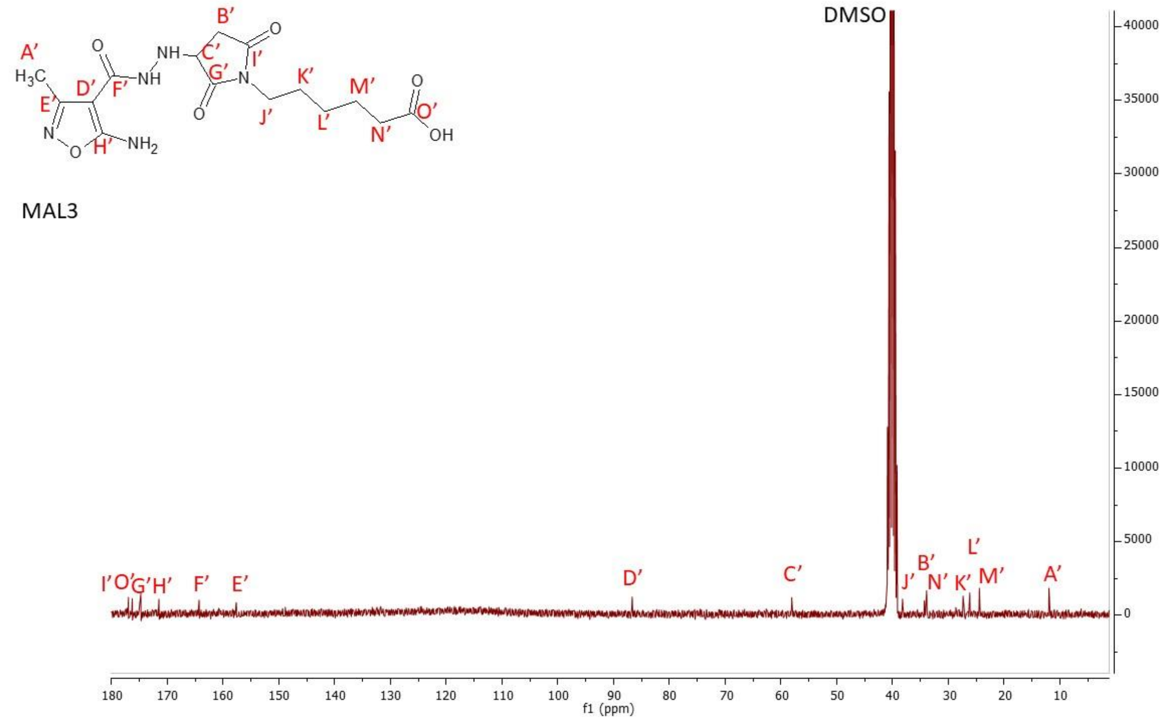

Figure S8. ^{13}**C NMR spectrum of compound MAL3**

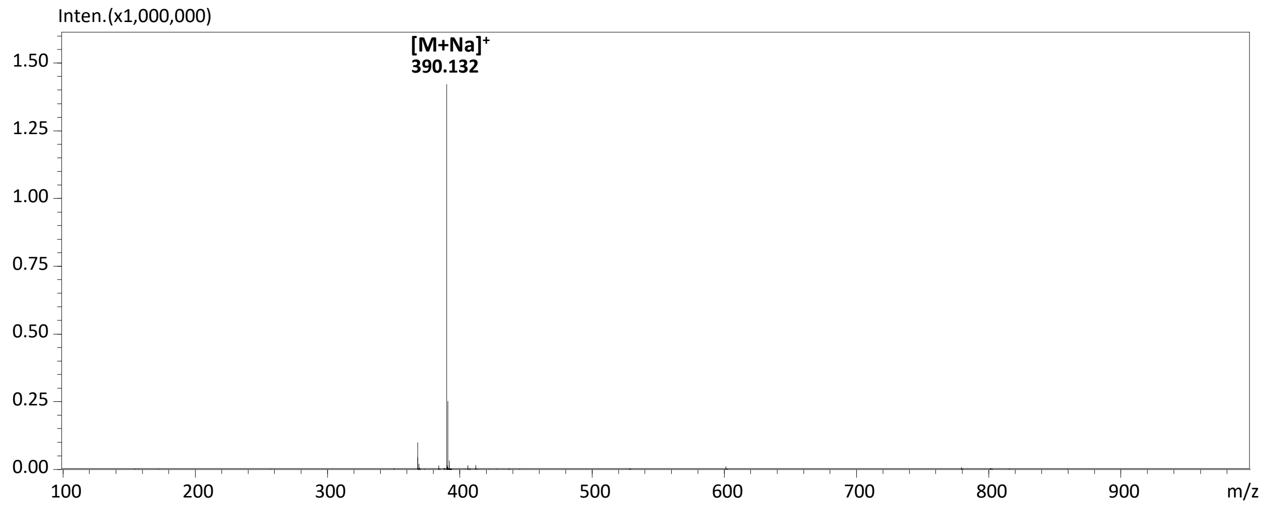

Figure S9. ESI-MS spectrum of compound MAL3

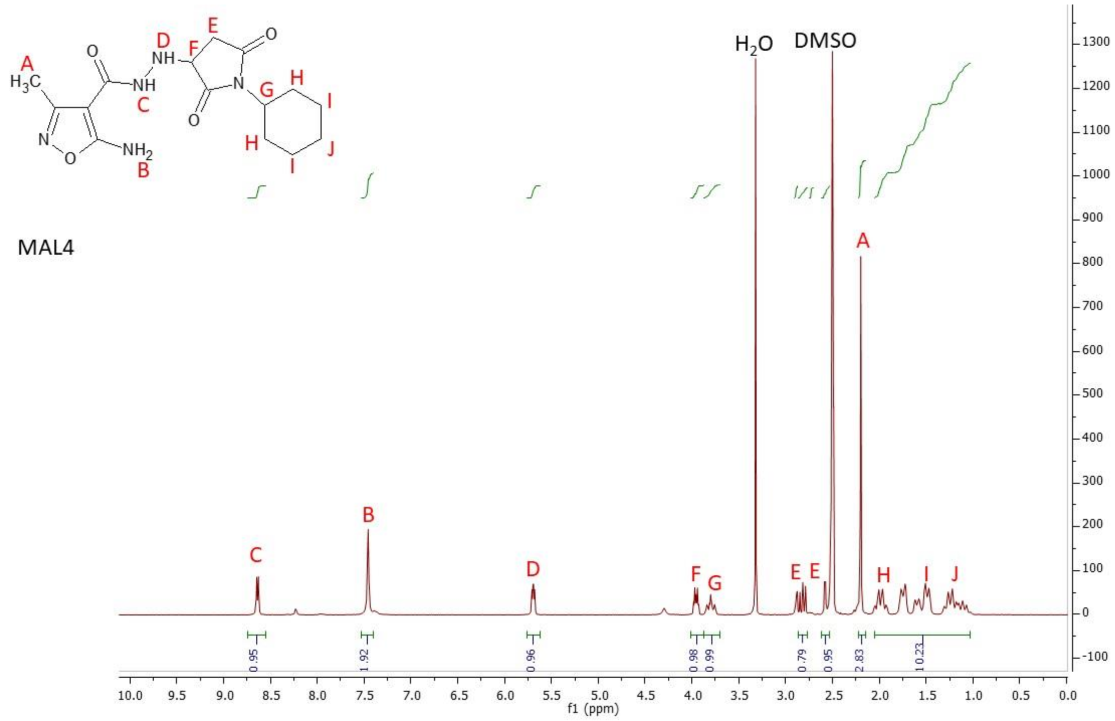

Figure S10. ¹H NMR spectrum of compound MAL4

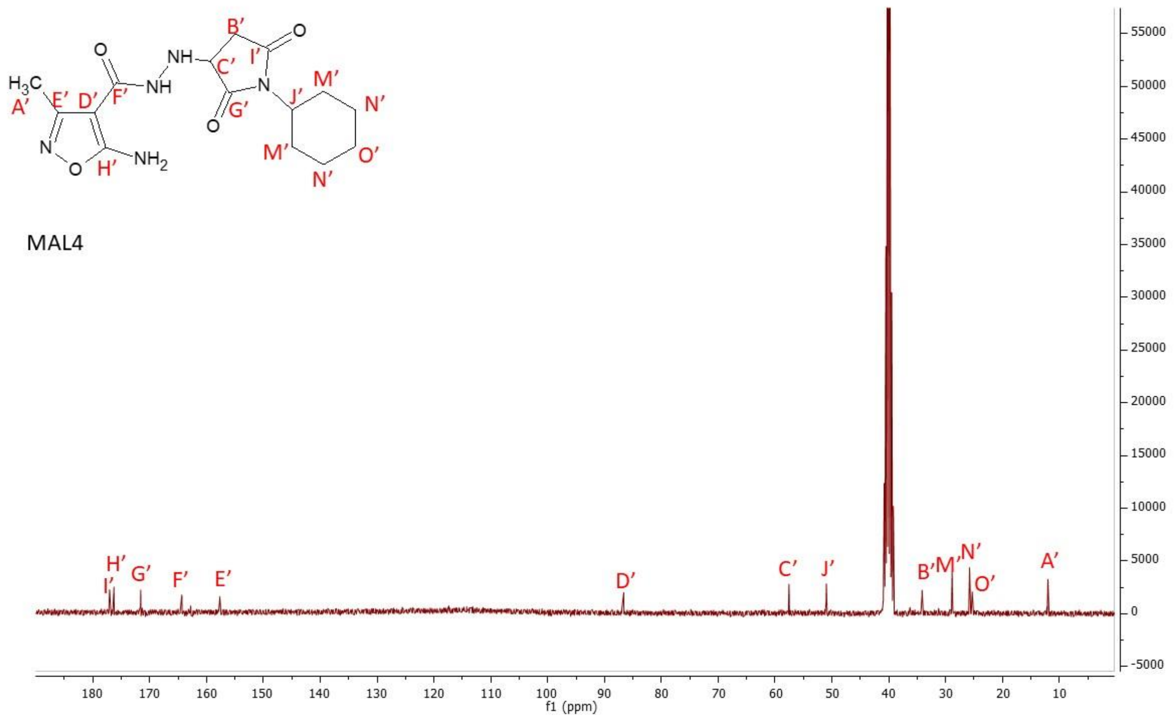

Figure S11. ^{13}C NMR spectrum of compound MAL4

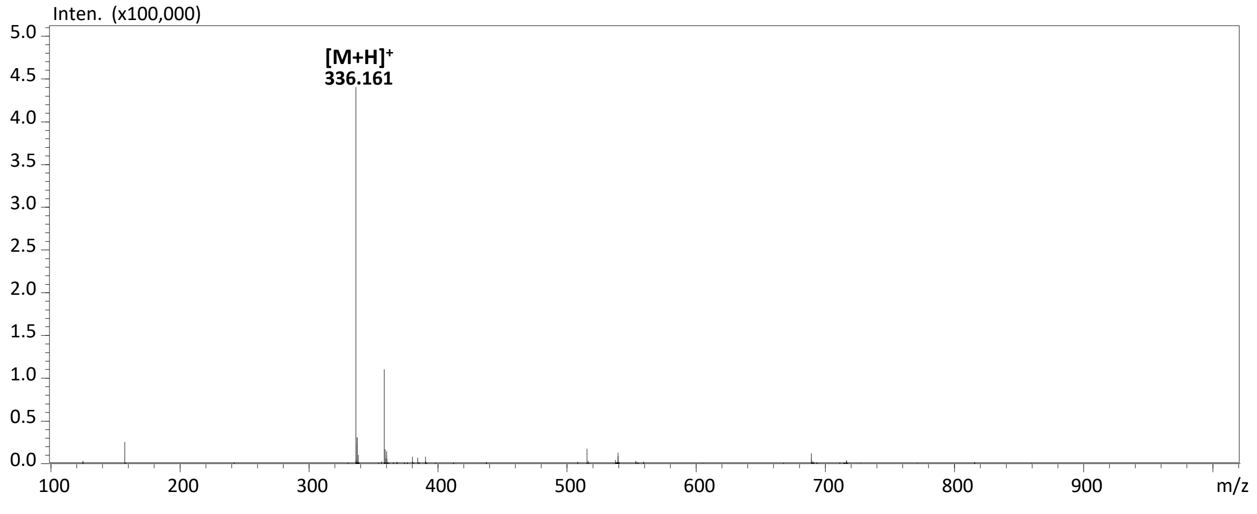

Figure S12. ESI-MS spectrum of compound MAL4

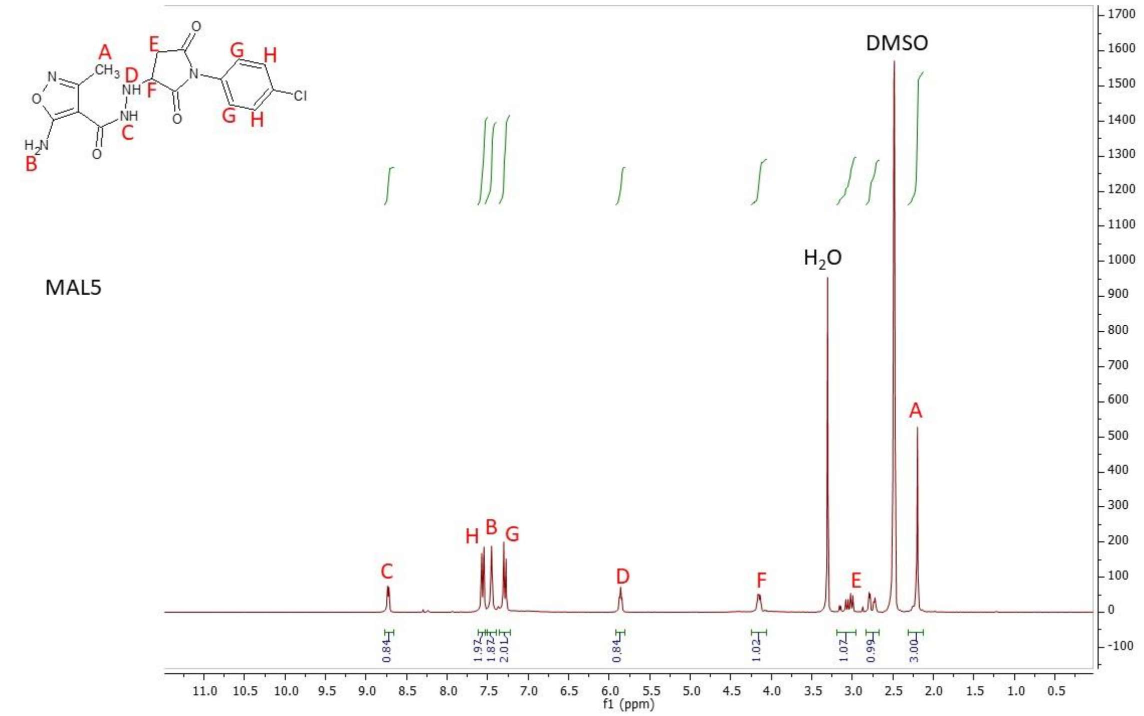

Figure S13. **^{1}H NMR spectrum of compound MAL5**

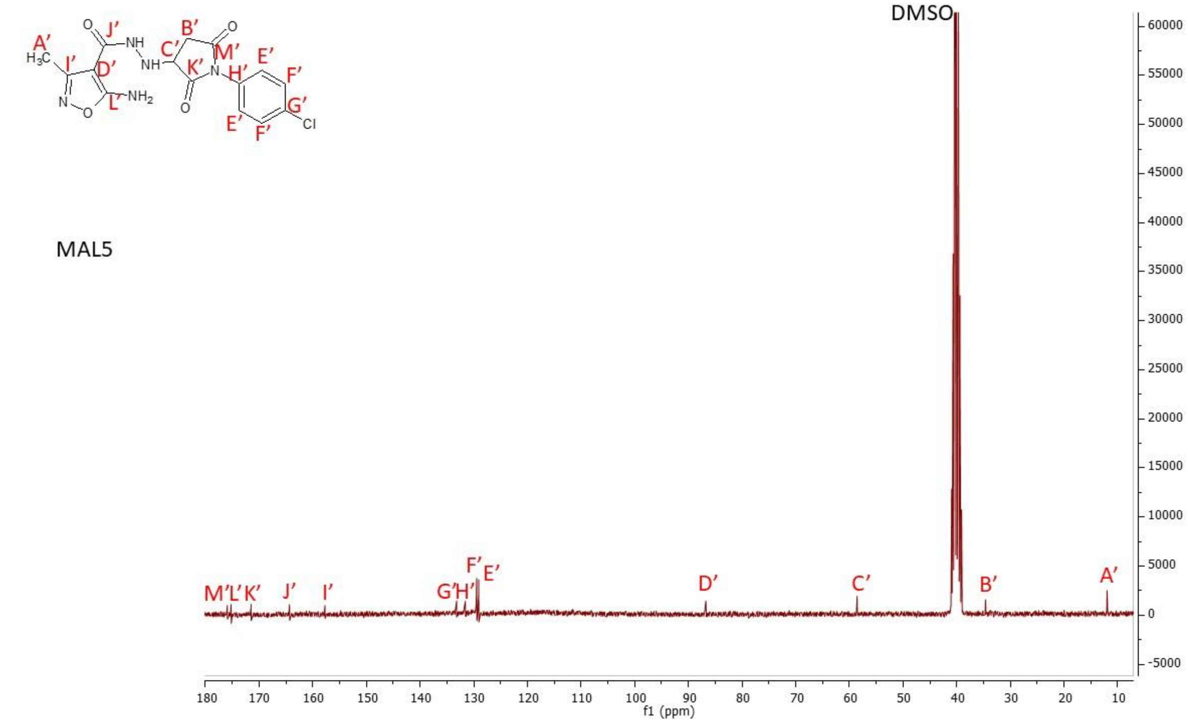

Figure S14. **^{13}C NMR spectrum of compound MAL5**

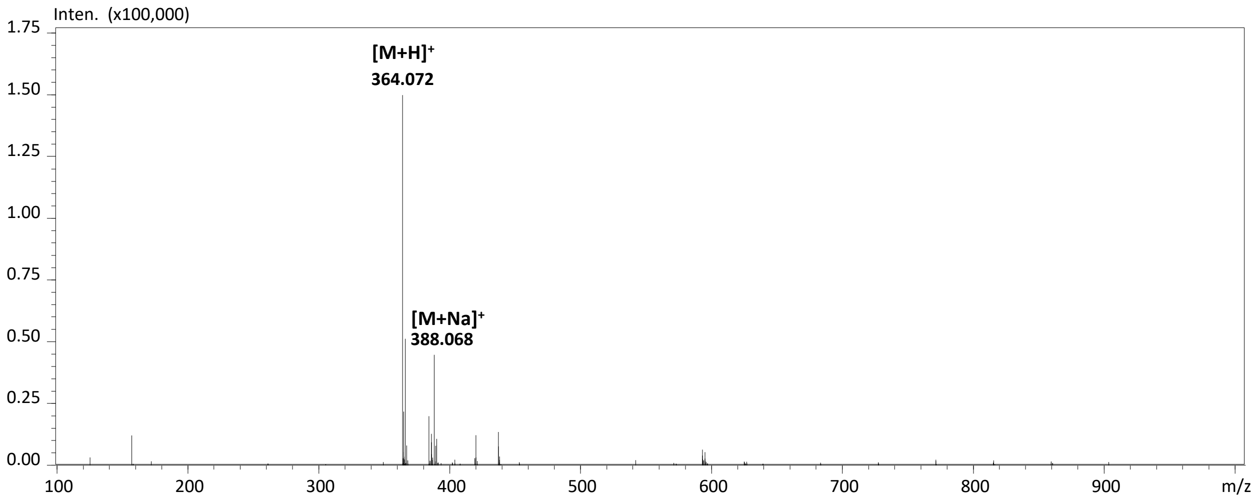

Figure S15. ESI-MS spectrum of compound MAL5

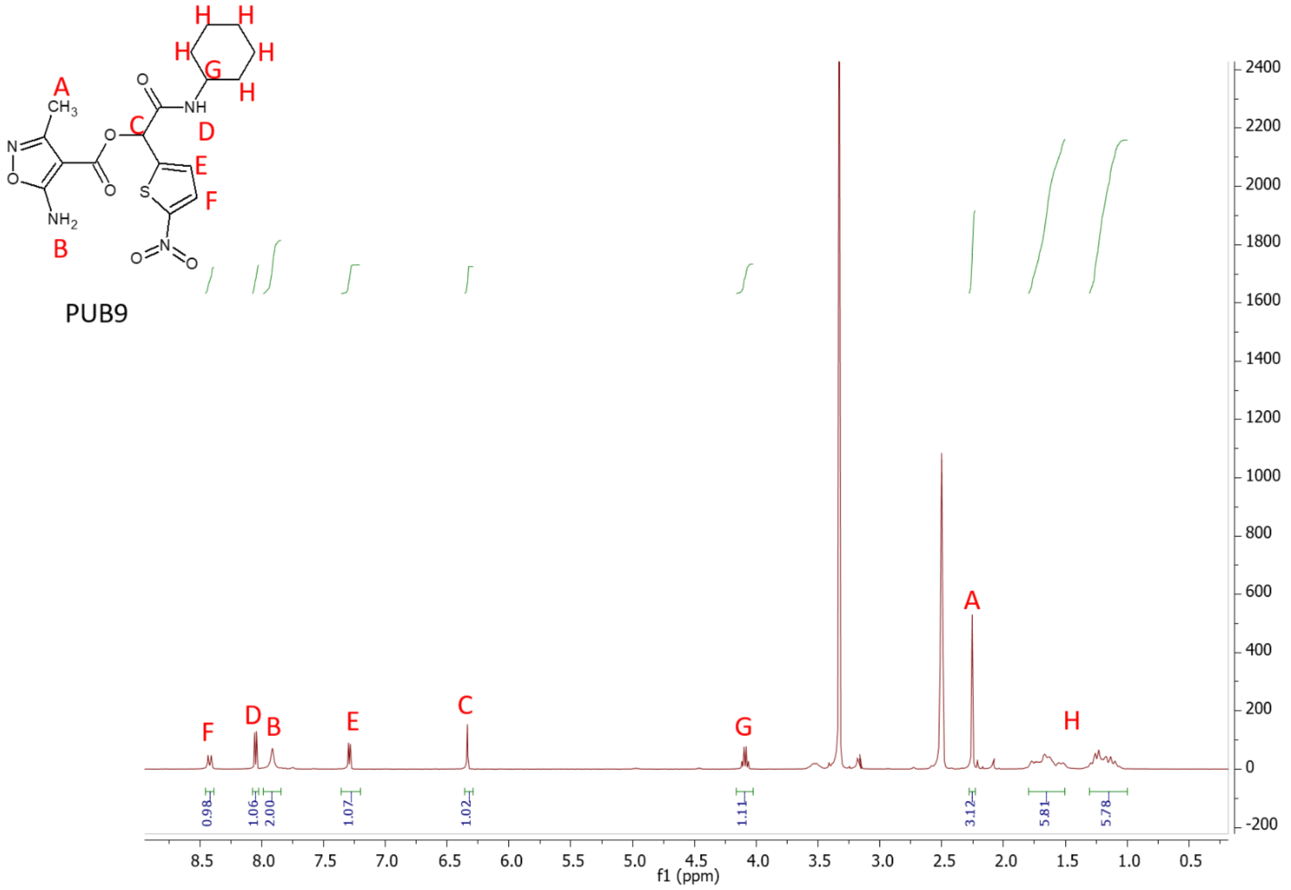

Figure S16. ^{1}H NMR spectrum of compound PUB9

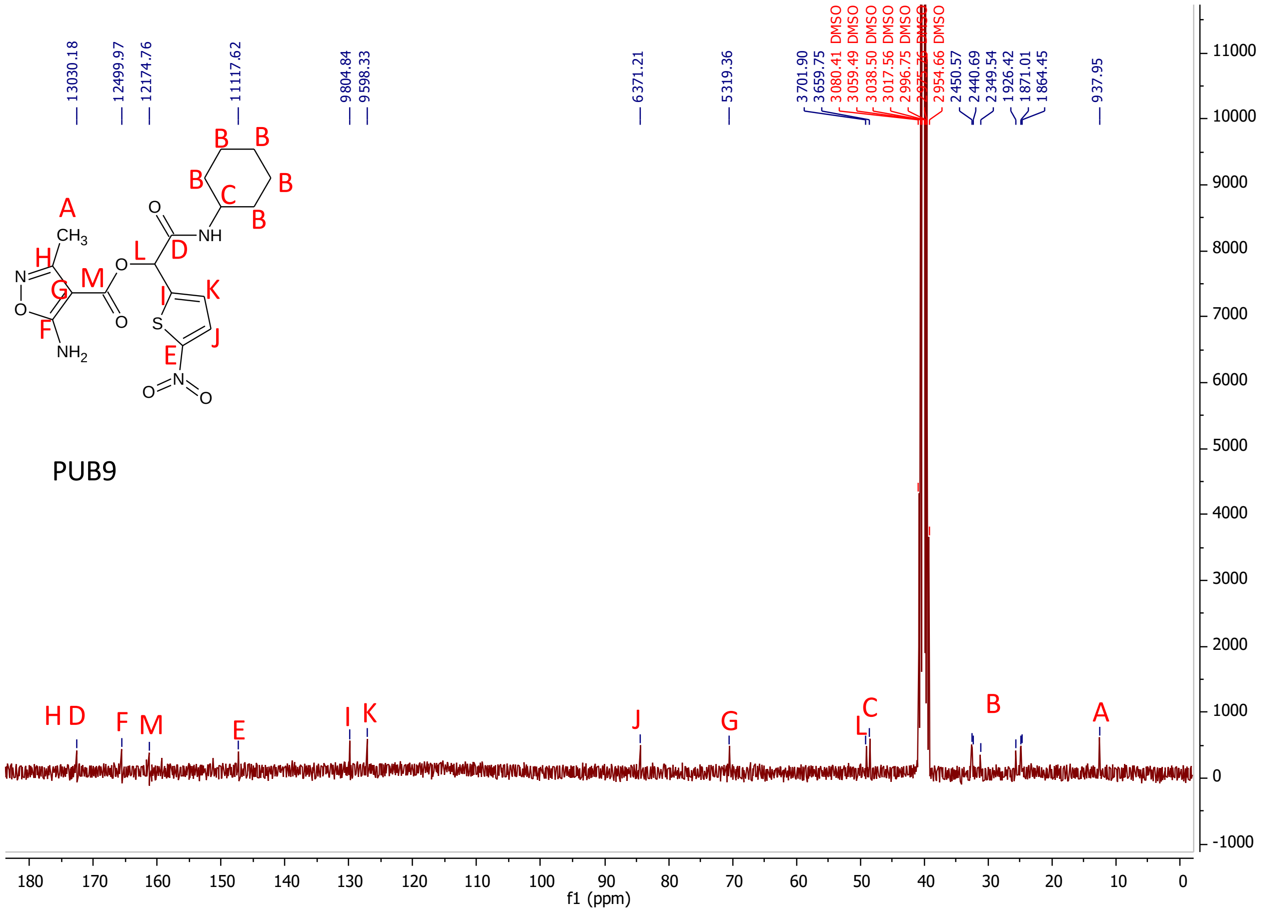

Figure S17. ^{13}C NMR spectrum of compound PUB9

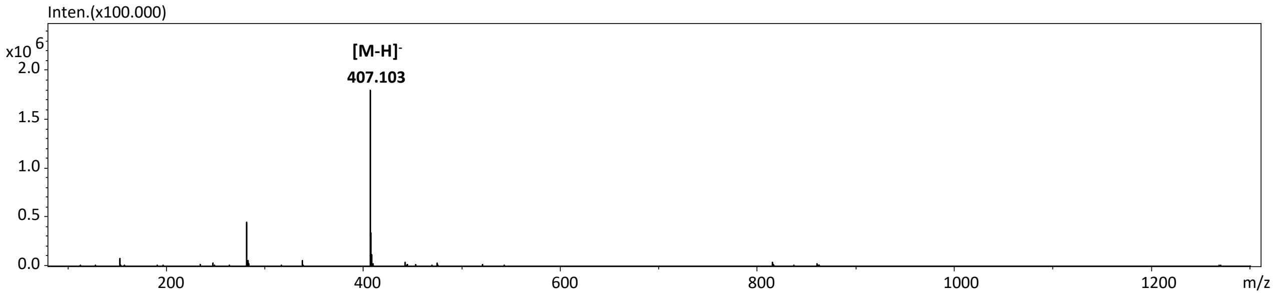

Figure S18. ESI-MS spectrum of compound PUB9

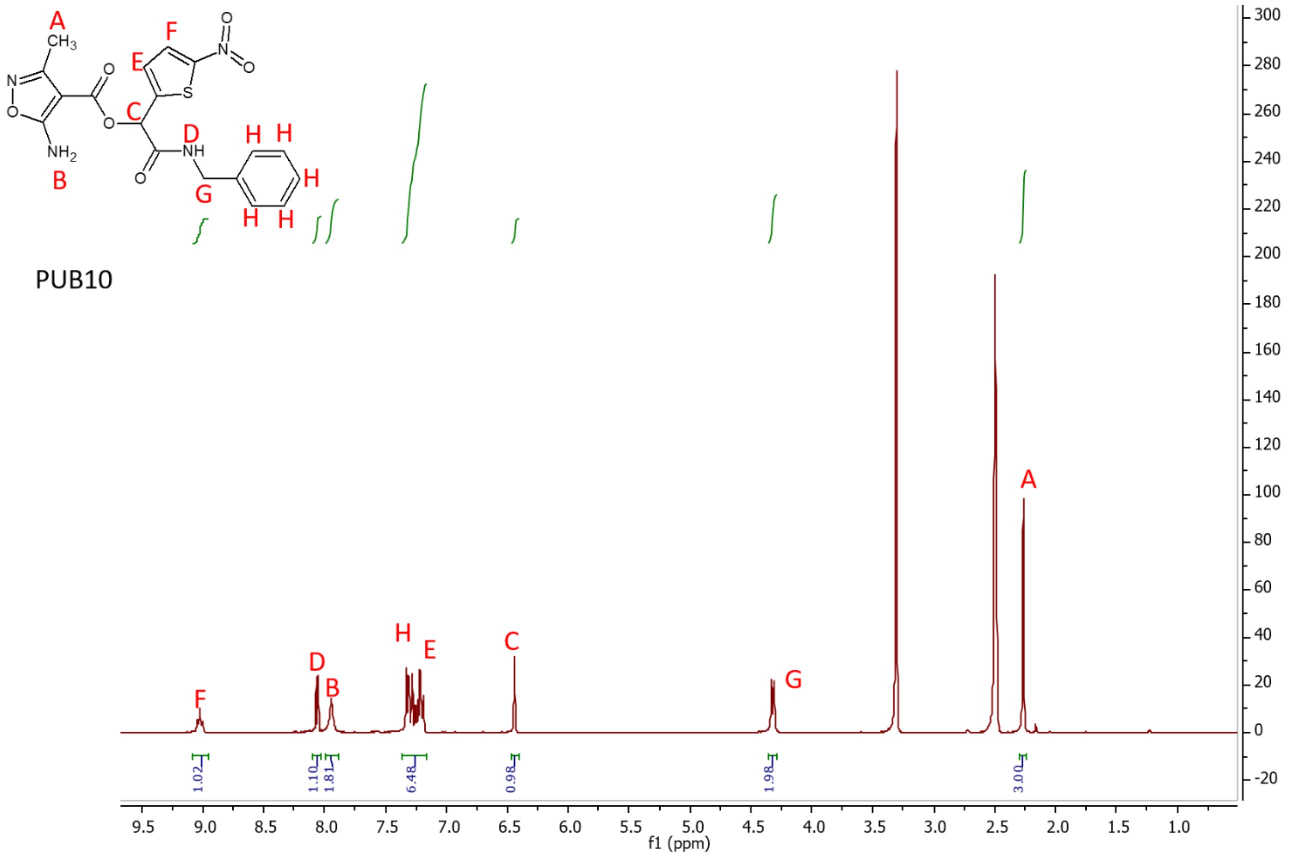

Figure S19. ¹H NMR spectrum of compound PUB10

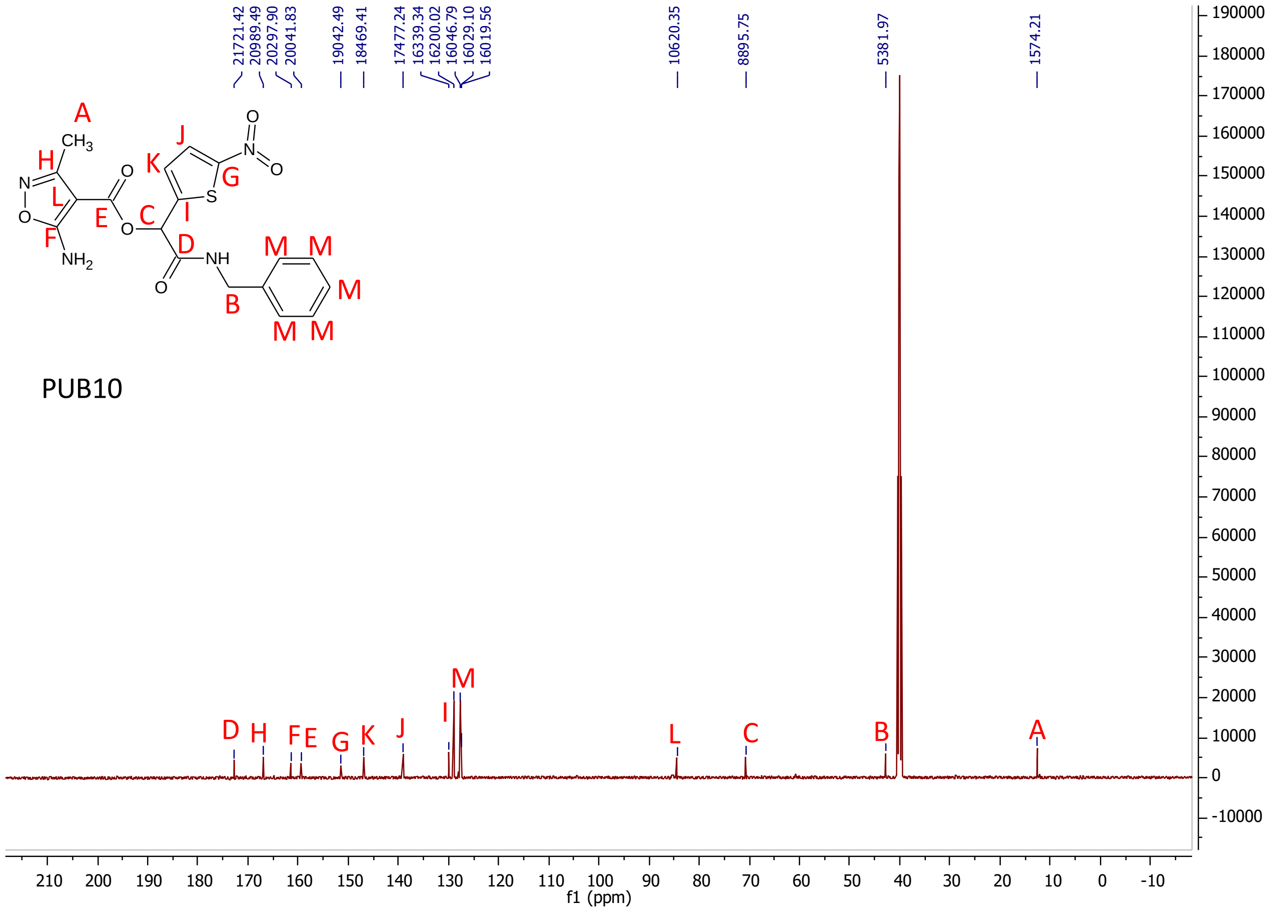

Figure S20. ¹³C NMR spectrum of compound PUB10

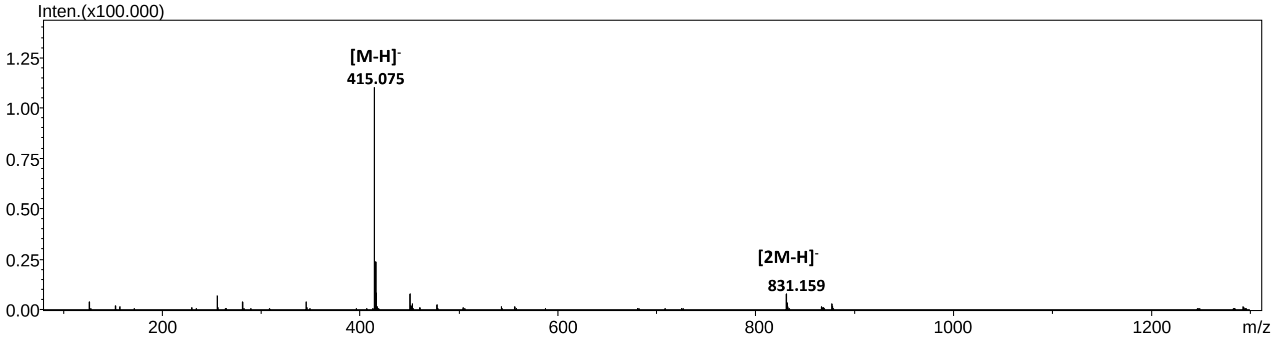

Figure S21. ESI-MS spectrum of compound PUB10

Table S1. The values of Minimal Inhibitory Concentration (MIC), Minimal Biofilm Eradication Concentration (MBEC) and Antibiofilm Dressing Activity Measurement (A.D.A.M.) performed for octenidine dihydrochloride-based antiseptic agent (method's usability control).

	S.aureus	***P.aeruginosa***	***C.albicans***
**MIC [mg/L]**	**1**	**0,78**	**1**
**MBEC [mg/L]**	**62,5**	**1000**	**62,5**
**A.D.A.M. [%]reduction]**	**98**	**95**	**89**
